# Complete, closed bacterial genomes from microbiomes using nanopore sequencing

**DOI:** 10.1038/s41587-020-0422-6

**Published:** 2020-02-10

**Authors:** Eli L. Moss, Dylan G. Maghini, Ami S. Bhatt

**Affiliations:** 1 Department of Genetics, Stanford University, Stanford, CA USA; 2 Department of Medicine (Hematology, Blood and Marrow Transplantation), Stanford University, Stanford, CA USA

**Keywords:** Microbiology, Metagenomics, Genome informatics, Genome

## Abstract

Microbial genomes can be assembled from short-read sequencing data, but the assembly contiguity of these metagenome-assembled genomes is constrained by repeat elements. Correct assignment of genomic positions of repeats is crucial for understanding the effect of genome structure on genome function. We applied nanopore sequencing and our workflow, named Lathe, which incorporates long-read assembly and short-read error correction, to assemble closed bacterial genomes from complex microbiomes. We validated our approach with a synthetic mixture of 12 bacterial species. Seven genomes were completely assembled into single contigs and three genomes were assembled into four or fewer contigs. Next, we used our methods to analyze metagenomics data from 13 human stool samples. We assembled 20 circular genomes, including genomes of *Prevotella copri* and a candidate *Cibiobacter* sp. Despite the decreased nucleotide accuracy compared with alternative sequencing and assembly approaches, our methods improved assembly contiguity, allowing for investigation of the role of repeat elements in microbial function and adaptation.

## Abstract (web summary)

Nanopore-metagenomics workflow enables assembly of complete, closed bacterial genomes from human stool samples.

## Main

De novo generation of finished metagenome-assembled genomes (MAGs) for bacteria and archaea is a longstanding goal in microbiome research. As existing metagenomic sequencing and assembly methods do not usually yield finished bacterial genome sequences, genome drafts are formed by grouping or ‘binning’ similar contigs. This approach has produced enormous collections of bacterial genomes and substantially expanded our appreciation of the microbial world^1–4^.

Binning quality largely relies on the size and contiguity of the underlying assembly. As assembly contiguity increases, the sensitivity and specificity of genome binning are improved, because fewer, larger contigs need to be grouped to form each genome. Advances in sequencing and assembly technologies, including read-cloud sequencing, have improved MAG quality^5^, but remain limited in their ability to correctly place repeat sequences.

Repeat elements can range in size from tens of base pairs to hundreds of kilobases^6^. Long reads can span entire common repeat elements such as miniature inverted repeat transposable elements, transposons, gene duplications and prophage sequences. Recently, nanopore and PacBio long-read assembly methods have been applied to the gut and other microbiomes^7,8^. However, the application of long-read methods to analyze gut microbiomes has been hindered by the lack of efficient methods to extract high molecular weight (HMW) DNA from stool. Standard bead beating can result in extensive shearing, and although solid phase reversible immobilization (SPRI) bead ‘cleanup’ steps remove DNA fragments in the low hundreds of base pairs, this often fails to enrich for DNA fragments that are sufficiently large to scaffold across bacterial repeat elements. Gentle bead beating can reduce shearing, but might fail to extract DNA from organisms that are difficult to lyse. Thus, there is a need for methods to extract long fragments of DNA that can span repetitive elements from both Gram-positive and Gram-negative bacteria to overcome limitations in genome assembly^6^.

We present a workflow for nanopore sequencing of stool samples, including protocols for DNA extraction and genome assembly (Supplementary Fig. 1). Our DNA extraction protocol is adapted from extraction methods for cultured bacteria^9^, and comprises enzymatic degradation of the cell wall with a cocktail of lytic enzymes, then phenol-chloroform extraction, followed by RNAse A and Proteinase K digestion, gravity column purification and SPRI size selection. This approach produces microgram quantities of pure, HMW DNA suitable for long-read sequencing from as little as 300 mg of stool. Our bioinformatics workflow, Lathe, uses a long-read assembly based approach, rather than a hybrid assembly method such as OPERA-MS, which was recently reported^8^. Input long-read data can be generated by either nanopore or PacBio technologies. Lathe combines existing steps for basecalling, long-read assembly and polishing with refined approaches for misassembly detection and genome circularization (Methods).

**Fig. 1:**
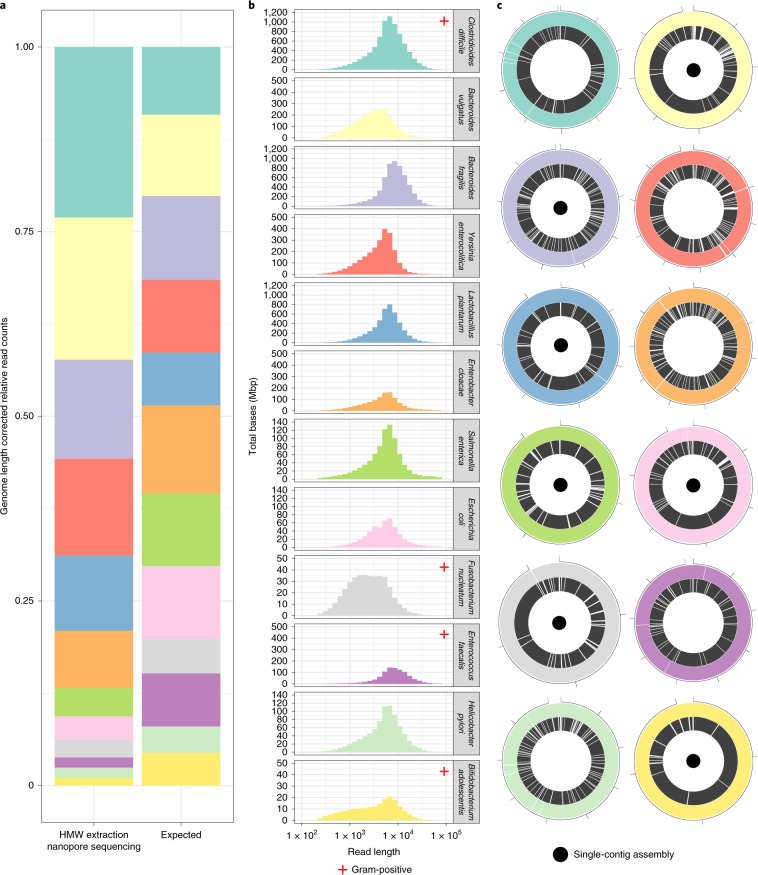
Taxonomic read composition, per-organism read-length distributions and genome assemblies in a defined 12-species bacterial mixture. **a**, Relative read counts are shown for the expected equal composition of bacterial cells and the observed composition, with correction for relative genome size. **b**, Read-length distributions per organism. Individual organisms demonstrate varying read-length distributions in some cases. **c**, Circos plots demonstrate the relative assembly contiguity of the nanopore versus short-read assembly approaches. Nanopore sequencing and assembly (colored outer ring) outperforms short-read assembly (black inner ring), producing complete genome assemblies (small black inner dots) in seven of 12 cases, with a further three assembled in four contigs or fewer. Numbers indicate genome size in megabases. Note that complete assemblies may contain one apparent break due to differing linearization breakpoints in reference and assembly sequences.

We first sought to test whether we could assemble closed bacterial genomes by using a standard ATCC 12-species mixture (Methods) that comprises both Gram-positive and Gram-negative bacteria. Due to the low concentration of HMW DNA present in the lyophilized cell material provided and the reduced contamination in this synthetic mixture compared with stool samples, we omitted the digestion and gravity column steps for DNA extraction (Methods) and obtained 401 ng of HMW DNA (Supplementary Table 1 and Supplementary Fig. 2). We used nanopore sequencing and obtained 30.3 gigabase pairs (Gbp) of long-read data with a read N50 of 5.9 kilobase pairs (kbp) (Supplementary Table 2) containing all 12 constituent species in approximately even relative abundances after correction for genome length (Fig. 1). Taxonomic classification of long reads demonstrated that read-length distributions vary between organisms (Fig. 1) from a minimum read N50 of 2.3 kbp (*Fusobacterium nucleatum*) to a maximum of 8.5 kbp (*Bacteroides fragilis*), perhaps a consequence of slight variations in response to lysis, extraction, lyophilization or storage. Gram-positive bacteria (red crosses, Fig. 1), which generally have a thicker peptidoglycan cell wall than Gram-negative bacteria, are not categorically depleted in relative abundance.

Assembly using Lathe yielded a total assembly N50 of 4.6 Mbp and total length of 48 Mbp, in agreement with the known total reference length (Supplementary Table 3). Lathe achieved a higher assembly N50 than other long-read assembly tools (1.6- to four-fold improvement) and hybrid assembly tools (two- to nine-fold improvement) (Supplementary Table 4). By contrast, assembly with SPAdes^10^ of a 7.7 Gbp public short-read dataset from the same mixture produces an assembly N50 of 133 kbp. Assembly with Lathe of nanopore data, randomly downsampled to equal the smaller total size of the short-read dataset, yielded an N50 of 3.3 Mbp, a 25-fold improvement over the short-read assembly.

Of the 12 bacteria in the mixture, seven were assembled into single contigs and shown to be complete by alignment to available closed reference sequences (Fig. 1). Three more genomes were assembled into four contigs or fewer. The most incomplete assembly contained 83% of the genome in a single contig. Our assembly contains large-scale inversions in *Bacteroides vulgatus* and *Enterobacter cloacae* relative to reference genome sequences. These were retained after multiple misassembly removal steps as the assembled inversion breakpoints were each spanned by multiple long reads. These inversions are flanked by homologous regions >20 kbp in length; this makes determining the true orientation of the inverted segment extremely challenging and, thus, we are unsure whether this represents an error in our assembly or the reference sequence. MetaQuast^11^ comparison of available closed reference genome sequences to our assemblies after consensus refinement with long reads, short reads or combined long and short reads indicates that short-read refinement is sufficient for error correction (Supplementary Note 1), but we have included the option of combining both forms of correction for cases of sparse short-read coverage in the Lathe workflow.

Next, we applied our methods to two human stool samples that were previously used to evaluate short-read and read-cloud sequencing and assembly approaches^5^, here referred to as samples P1 and P2-A, as well as a stool sample collected 15 months after the first sample from individual P2; we refer to this second sample as P2-B. Our extraction approach yielded at least 1 µg of pure HMW DNA per 300 mg of input stool mass for all samples (Supplementary Table 1). We tested the potential generalizability of the extraction approach on canine and murine stool samples and obtained similar yield, purity and fragment size across all samples tested (Supplementary Fig. 2).

**Fig. 2:**
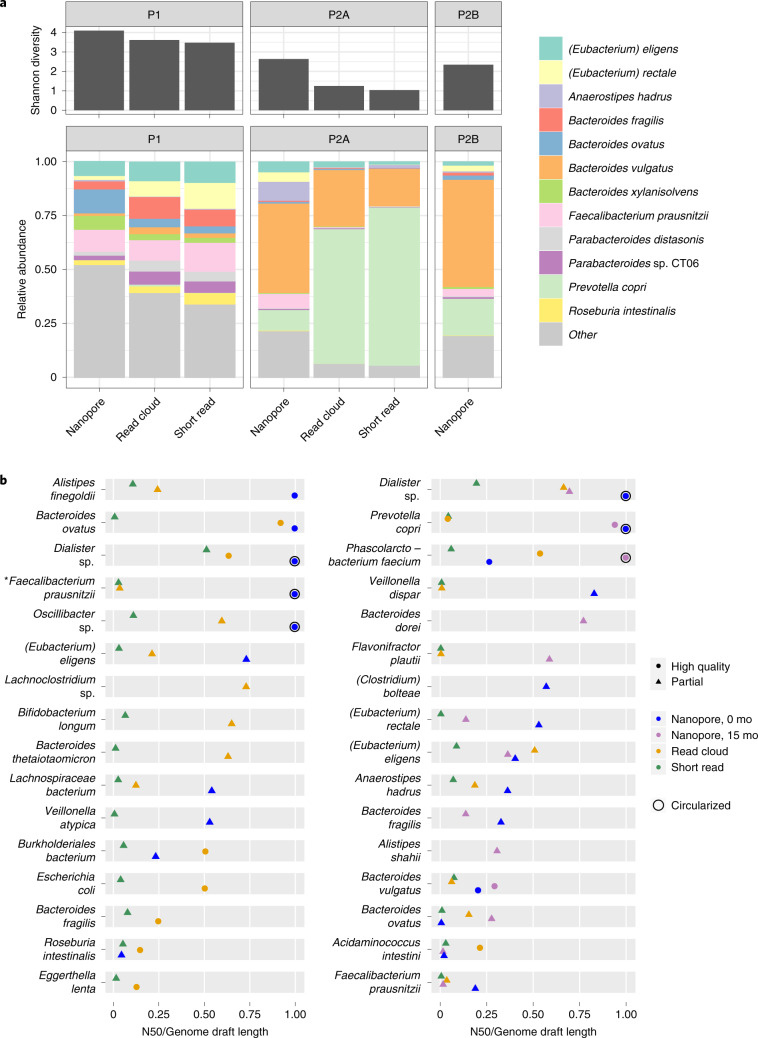
Per-organism assembly contiguity, diversity and taxonomic read composition in two healthy human stool microbiomes. **a**, Species-level Shannon diversity is shown for the sequence datasets obtained. Higher diversity is found in libraries prepared with the present DNA extraction method. Relative species-level abundances are shown for a conventional workflow consisting of bead-beating extraction and short-read sequencing, as well as the present workflow consisting of HMW DNA extraction and long-read sequencing. **b**, Contiguity is expressed as per-bin N50 divided by per-bin length (the total length of sequences assigned to the bin). As bin assembly approaches completion, the quantity N50 divided by bin length approaches one, regardless of genome size. Nanopore sequencing and assembly (blue, purple) demonstrates higher assembly contiguity than read-cloud (gold) and short-read (green) approaches. For all organisms achieving assembly N50 of at least 500 kbp or a complete genome draft by any approach, genome draft quality and contiguity are shown for long reads, read clouds and short reads. Shapes indicate draft quality. Asterisk marks a genome later annotated as putative *Cibiobacter*.

After nanopore sequencing, we obtained a total of 12.7, 6.1 and 7.6 Gbp of long-read data for samples P1, P2-A and P2-B, respectively (Supplementary Table 2) with read N50 values of 4.7, 3.0 and 3.0 kbp (Supplementary Table 2 and Supplementary Fig. 3). DNA from these samples was extracted before the incorporation of MetaPolyzyme into our approach, so it is possible that taxa that are difficult to lyse may be underrepresented. Nonetheless, taxonomic composition of reads obtained through this version of our approach had higher Shannon diversity when compared with reads from samples extracted with mechanical lysis and short-read sequencing (Fig. 2). Specifically, we recovered all genera detected by more than 200 short reads and there was no categorical depletion of typically lysis-resistant Gram-positive organisms (Supplementary Fig. 4). Per-species read-length distribution for organisms occurring in the natural samples was less variable than in the synthetic bacterial mixture (Supplementary Fig. 5). We observed a prominent 3–4 kbp subset of reads classified as *Escherichia coli* in all samples and a second 12–15 kbp subset of reads classified as *Enterococcus faecalis* in the defined bacterial mixture (Supplementary Fig. 5). These reads originate from sequences with high identity to phage and are not found in a PacBio library prepared from the same extraction, suggesting contamination of nanopore libraries with phage DNA.

Assembly with Lathe yielded whole-assembly N50 values of 236, 221 and 179 kbp and total assembly sizes of 139, 83 and 87 Mbp for samples P1, P2-A and P2-B, respectively. Employing a strategy to improve metagenomic assembly of related communities^12^, we coassembled samples P2-A and P2-B and obtained a 1.7-fold increase in assembly N50 (384 kbp) and a 1.3-fold increase in total assembly size (112 Mbp) (Supplementary Table 5). In comparison, short-read assembly yielded assembly N50 values of 34 and 15 kbp for P1 and P2-A, in spite of a three- to six-fold higher input (total bases) of raw read data, and read-cloud assembly yielded N50 values of 116 and 12 kbp in P1 and P2-A. However, read-cloud and short-read assemblies were between 1.5- and 2.1-fold larger in total than corresponding nanopore long-read assemblies, likely due to the much greater volume of raw data in these datasets (Supplementary Tables 2 and 5). Sequencing with PacBio produced an assembly that was much more fragmentary than that produced by nanopore sequencing and assembly with Lathe, likely due to more variable coverage with PacBio sequencing (Supplementary Note 2).

After binning contigs from nanopore, read-cloud and short-read approaches to form draft genomes^5^, drafts were scored as ‘High Quality’ or ‘Partial’ based on completeness, contamination, and presence of 5S, 16S, 23S ribosomal RNA and transfer RNA loci^1^. Completeness and contamination were assessed using checkM^13^, a tool that evaluates for presence of single-copy core genes; while broadly applied and useful, circumstances have been documented where estimates of completeness and contamination are inaccurate^14^. The long-read approach produced bins with much higher contiguity than the read-cloud approach, at lower cost, (Fig. 2, Supplementary Fig. 6 and Supplementary Table 6), yielding several high-quality genomes with N50 over 2 Mbp, whereas the read-cloud approach yielded only one and the short-read approach yielded no bins with N50 values greater than 0.55 Mbp. Nanopore sequencing assembled several single-contig, high-quality genomes from each sample, including genomes for *Dialister* sp., *Faecalibacterium prausnitzii*, *Oscillibacter* sp. and *P. faecium*, all of which had fragmentary read-cloud and short-read assemblies (Fig. 2 and Supplementary Fig. 7). Notably, our approach produced a circular genome for *P. copri*, an organism that lacked a closed reference until recently^7^, in spite of extensive previous efforts to assemble it and other members of the genus^15^. Several bins in the P1 read-cloud assembly were absent from nanopore bins, likely due to their low coverage depth (3–40×), resistance to lysis or cooccurrence with closely related community members.

We then sought to evaluate the generalizability of our extraction and nanopore sequencing approach and to test whether this HMW DNA extraction approach generated taxonomically concordant results compared to conventional bead beating. With MetaPolyzyme incorporated in the lysis stage, we applied the two extraction approaches to ten additional stool samples from healthy adults (samples A–J). An adequate amount of size-selected DNA was obtained from all ten of the samples using the HMW DNA extraction approach. While we attempted to obtain sufficiently size-selected DNA for nanopore sequencing from DNA extracted with bead beating for a subset of the stool samples, we were only able to obtain adequate DNA from one sample (Supplementary Fig. 8). Nanopore sequencing of the HMW extracted DNA yielded 13 to 27 Gbp of raw long-read data with read N50 values ranging from 1.4 to 5.2 kbp, which was combined with comparatively light coverage of 1.9 to 3.6 Gbp of short-read data for consensus refinement and determining taxonomic composition (Supplementary Table 2). Nanopore sequencing on the sample that had sufficient bead-beaten DNA yielded a read N50 of 2.5 kbp and 6.3 Gbp of data, compared to 2.7 kbp and 15.9 Gbp for nanopore sequencing of a HMW extraction on the same sample. Both extraction methods yielded similar taxonomic compositions when nanopore sequenced (Supplementary Fig. 9). Nanopore and short-read data were classified and compared across samples (Supplementary Figs. 10 and 11 and Supplementary Table 7). On log-transformed read counts of all 596,300 species classified, we measured an overall correlation (Pearson *r* = 0.79) between the two approaches. The number of read counts ranged from 1 to 5.7 × 10^6^, with a mean of 620 (Supplementary Fig. 12). Of the 18,642 instances of a ten-fold or greater difference in relative abundance of a particular species between the two approaches, our approach yielded the higher relative abundance in 95% of cases, suggesting the potential for greater taxonomic sensitivity by our method. While 3,566 classifications made by the bead beating and short-read approach were undetected by the present approach, our approach made 72,989 classifications undetected by the bead-beating and short-read approach. Across the ten samples, we obtained assemblies ranging in total length between 48 and 207 Mbp and assembly N50 between 51 and 120 kbp, the two generally inversely correlated (Supplementary Table 5). This is slightly reduced compared to the assemblies obtained from human stool samples P1 and P2, likely due to our use of the Flye assembler in place of Canu, incurring much lower computational cost in exchange for a modest reduction in contiguity (see Methods and Supplementary Table 4). In situations where high contiguity is desired, such as attempts to generate a complete, closed genome of a novel taxon or when sensitive detection of structural variants or horizontally transferred intrachromosomal genomic regions is desired, Canu may be the preferred assembler. Alternatively, when aiming to obtain as many high-quality genome bins as possible and cost is a higher priority consideration, Flye may be the preferred assembler.

This sequencing and assembly approach is capable of generating closed, circular genomes (Table 1). However, as we learned from our sequencing and assembly experiments with the mock mixtures, fully assembled genomes often evade circularization from their initial linear form. In the context of mock mixtures, the ground truth is known; however, in natural samples where a ground truth is lacking, it is difficult to determine whether genomes that do not circularize are truly ‘full genomes’^14^.

**Table 1:** Circular bacterial genomes assembled from human stool samples

Genome	Sample	Assembler	Genome size (Mbp)	Genes	16S rRNA	GC percentage	Transposases^b^
*Dialister* sp.	P1	Canu	1.96	1,912	4	45.1	2
*Dialister* sp.	P2-A	Canu	1.89	1,803	4	45.3	7
*Faecalibacterium prausnitzii*^c^	P1	Canu	3.4	3,234	6	56.1	45
*Oscillibacter* sp.	P1	Canu	3.04	2,926	3	60.1	4
*Phascolarctobacterium faecium*	P2-B	Canu	2.35	2,307	5	44.0	0
*P. copri*	P2-A	Canu	3.71	3,324	5	45.0	69
*Akkermansia muciniphila*	I	Flye	3.01	2,906	3	55.2	0
*Anaerotruncus* sp.	F	Flye	2.11	2,156	2	43.5	4
*Bacteroides* sp.	F	Flye	3.04	2,467	3	48.9	18
*Clostridales* sp.^a^	D	Flye	2.05	1,971	2	53.2	0
*Eubacterium siraeum*^a^	F	Flye	3.12	2,894	3	45.3	12
*Eubacterium* sp.^a^	G	Flye	2.11	2,043	2	44.8	1
*Methanobrevibacter smithii*	B	Flye	1.78	3,579	2	30.9	1
*Oscillibacter* sp.^a^	G	Flye	3.29	3,169	3	59.6	18
*Phascolarctobacterium faecium*	I	Flye	2.35	2,481	5	43.4	0
*Prevotella* sp.	F	Flye	3.46	3,031	5	45.8	78
*Roseburia* sp.^a^	D	Flye	2.17	2,953	2	40.9	8
*Ruminococcus bromii*	G	Flye	2.21	2,820	3	40.7	20
*Ruminococcus* sp.	E	Flye	2.48	3,007	4	42.1	0
*Sellimonas intestinales*^a^	D	Flye	1.76	2,889	3	45.2	11

^a^Assembly broken into multiple contigs during final misassembly detection, perhaps due to off-by-one circularization.

^b^Transposases annotated with Prokka v.1.13.3.

^c^Later annotated as putative *Cibiobacter* sp.

For example, from the three stool samples that were assembled with Canu (P1, P2-A and P2-B), our approach yielded eight high-quality, single-contig bacterial genomes and a maximum of five from a single sample (P1), compared to zero from short-read and read-cloud approaches^5^. Lathe achieved precise circularization for five of these genomes. Closed genomes ranged in coverage depth between 75× (*Oscillibacter* sp.*)* and 785× (*P. copri*), and were largely structurally concordant and similar in sequence to existing published genome sequences (Supplementary Fig. 7), although in some cases, we note extensive strain divergence. For example, our closed *Dialister* sp. genome exhibits multiple large-scale inversions relative to the available reference concordant with previous read-cloud and short-read assemblies^5^.

**Fig. 3:**
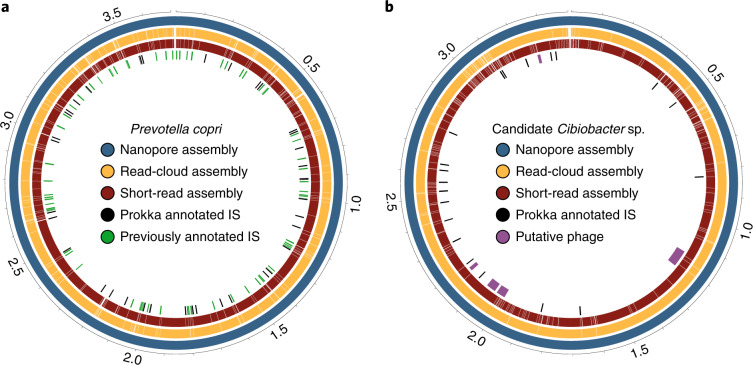
Circos diagrams of closed, circular genomes of *P. copri* and *Cibiobacter* sp. **a**, *P. copri* from P2-A. **b**, *Cibiobacter* sp. from P1. In both plots, the outermost ring represents the complete, closed and circularized genome of the given organism. The middle and inner rings represent contigs from the corresponding read-cloud and short-read assemblies, respectively, that were mapped to the nanopore assembly. The inner track in each case displays annotated, predicted mobile genetic elements such as insertion sequences (ISs), transposases and prophage.

The circular *P. copri* genome (Fig. 3a) is especially notable, as our own previous attempts using read clouds, short reads and synthetic long reads to assemble these communities also had limited success with this organism, never exceeding a genome N50 of 130 kbp in spite of attempts with coverage depth in excess of 4,800×, as well as with downsampled datasets^5^. While this is a report of a *P. copri* genome from a human sample, recently the first single-contig *P. copri* genome was reported using a nanopore approach on cow rumen^7^, supporting the use of longer reads in solving these difficult assemblies. The difficulty of assembling the *P. copri* genome stems from its high degree of sequence repetition. Previous assembly of repetitive *k*-mers in *P. copri* identified five repeat sequences with high identity to known transposase sequences^5^, and additional annotation with Prokka^16^ reveals additional insertion sequence transposases. The location of these high copy number elements is resolved in our circular assembly, and often fall at the locations of breaks in previous short-read and read-cloud assemblies of *P. copri*.

Noting high strain divergence between our circular *F. prausnitzii* genome and available references, we attempted to improve classification using 16S rRNA gene classification. Top hits for all six 16S rRNA sequences fell between *Gemmiger formicilis* (average of 98.11% identity) and *Subdoligranulum variabile* (average of 98.19% identity), compared to only 92.63% identity with *F. prausnitzii* type strain 16S rRNA sequences, indicating that this genome may be a member of the recently described *Cibiobacter* clade^17^ and may represent a closed genome for this genus (Fig. 3b). We identified five putative phage in the closed assembly, ranging in length from 8.5 to 65.9 kb.

Additionally, we obtained a total of 11 fully circularized, single-contig genomes from the set of ten samples that were assembled with Flye (samples A–J, Table 1). Among these is another completed *Prevotella* genome belonging to a species closely related to CAG:386 (92% draft aligned at 98% identity) representing a completed reference for this species. In total, we obtained 19 high-quality genomes, 16 of which had an N50 of over 1 Mbp and 12 had N50 over 2 Mbp (Supplementary Fig. 13). An additional 22 genomes met these criteria with minimum completeness reduced to 75%. In total, 1,219 genome drafts with contamination <5% were recovered, ranging in completeness from 0.31 to 100% with a mean of 23%.

However, even well covered genomes can fail to assemble when they have high identity with other organisms in the community, as Lathe cannot construct an unambiguous representation of a single contiguous genome from a highly interconnected assembly graph (Supplementary Fig. 14). Additionally, assembly contiguity remains closely tied to the DNA fragment length, and while the high read lengths achieved by our approach improve assembly contiguity over short-read approaches, further improvement in extraction methods will be necessary to achieve the longer read lengths needed to fully resolve clusters of highly related genomes and longer structural variants. Our extraction method has been demonstrated to more reliably extract HMW DNA from samples than bead-beating approaches do, thus offering many advantages for completing and circularizing genomes, but it does have extraction biases for different bacterial species that will necessitate further investigation. Gentler bead-beating approaches may also yield HMW DNA, but at the expense of potentially failing to extract DNA from difficult to lyse organisms. Therefore, standard bead beating remains the best approach for accurately measuring relative abundances of taxa. As short-read polishing improves assembly quality (Supplementary Note 1), reads used for polishing can inform relative abundance.

In the past several years, assorted molecular and computational approaches have been described for generating more complete genomes from metagenomes. Read-cloud sequencing with Athena assembly is advantageous in situations where DNA is scarce, as the input requirement is ~100-fold lower than that required for standard long-read sequencing approaches. This can be particularly helpful when studying low biomass samples, such as clinical samples. At present, limited comparisons between hybrid assemblers and long-read assembly followed by short-read polishing have been made. Based on the concepts that underlie these two approaches, hybrid assembly may be preferred in situations where low-coverage long-read sequence data is available.

In conclusion, we anticipate that our approach will enable the mapping of horizontally transferred gene segments, such as prophage, into specific genomic contexts. This may help to illuminate how structural strain variation within the microbiome may link to microbial function^18^. Furthermore, this approach enables the proper placement of repetitive genetic elements, as exist in the genus *Prevotella*, where such variations can be important in bacterial metabolic phenotypes^19^. Improved references in this group and elsewhere will facilitate study of diverse gut microbiomes across global populations by allowing investigation into the complete functional repertoire and potential phenotypes of individual microbes, even when these organisms are difficult to culture or occur in complex communities. We expect that advances in metagenomic DNA extraction methods, long-read sequencing, assembly algorithms and epigenetic modification detection^20^ will further improve the quality of MAGs, causing a profound shift in the effectiveness and resolution of metagenomic assembly.

## Methods

### Subject recruitment

Two healthy adult volunteers were recruited at Stanford University under an IRB-approved protocol (principal investigator, A. Bhatt). Ten healthy adults were recruited at Stanford University as a part of one of two IRB-approved protocols for tissue biobanking (PIs, A. Bhatt and V. Henderson). Informed consent was obtained for all human subjects. Animal stool samples were collected from a Great Pyrenees housepet dog and an 8–10-week-old female Balb/c(J) mouse obtained from the Jackson Laboratory. The discarded mouse stool samples were obtained as a part of an approved laboratory animal use protocol (PI, S. Artandi). We have complied with all relevant ethical regulations.

### Sample processing

Stool samples were placed at 4 °C immediately on collection, and processed for storage at −80 °C the same day. Stool samples were aliquoted into 2-ml cryovial tubes with no preservative. Samples were stored at −80 °C until extraction.

### Stool DNA extraction

Short-read and read-cloud libraries were prepared as previously described^5^. Briefly, DNA was extracted from samples P1 and P2-A with the Qiagen Stool Mini kit using standard bead-beating mechanical lysis. For read-cloud libraries, this was then size selected at 10 kbp with a BluePippin (Sage Science).

For HMW extraction, approximately 0.7 g of frozen stool was aliquoted into 2-ml Eppendorf tubes (Eppendorf) with a 4-mm biopsy punch (Integra Miltex and suspended in 500 µl of PBS (Fisher Scientific) with brief gentle vortexing. Then, 5 µl of lytic enzyme solution (Qiagen) and, for the mock mixture and stool samples from the ten subject healthy adult cohort, 10 µl of MetaPolyzyme (Sigma Aldrich; reconstituted in 750 µl of PBS) was added and the samples were mixed by gentle inversion six times, then incubated for 1 h at 37 °C. Next, 12 µl of 20% (w/v) SDS (Fisher Scientific) was added with approximately 100 µl of vacuum grease (Dow-Corning) functioning as phase lock gel. Then, 500 µl of phenol-chloroform isoamyl alcohol at pH 8 (Fisher Scientific) was added, samples were gently vortexed for 5 s and centrifuged at 10,000*g* for 5 min with a Legend Micro 21 microcentrifuge (Fisher Scientific). The aqueous phase was then decanted into a new 2-ml tube.

Next, DNA was precipitated with 90 µl of 3 M sodium acetate (Fisher Scientific) and 500 µl of isopropanol (Fisher Scientific) for 10 min at room temperature. After inverting three times slowly, samples were incubated at room temperature for 10 min, then centrifuged for 10 min at 10,000*g*. The supernatant was removed and the pellet was washed twice with freshly prepared 80% (v/v) ethanol (Fisher Scientific). The pellet was then air dried with heating for 10 min at 37 °C or until the pellet was matte in appearance, and then resuspended in 100 µl of nuclease-free water (Ambion, Thermo Fisher Scientific). Next, 1 ml of Qiagen buffer G2, 4 µl of Qiagen RNase A at 100 mg ml^−1^ and 25 µl of Qiagen Proteinase K were added, the samples were then gently inverted three times, and then were incubated for 90 min at 56 °C. After the first 30 min, pellets were dislodged by a single gentle inversion.

One Qiagen Genomic-tip 20/G column per sample was equilibrated with 1 ml of Qiagen buffer QBT and allowed to empty by gravity flow. Samples were gently inverted twice, applied to columns and allowed to flow through. Three stool extractions were combined per column. Columns were then washed with 3 ml of Qiagen buffer QC, then DNA was eluted with 1 ml of Qiagen buffer QF prewarmed to 56 °C. Eluted DNA was then precipitated by addition of 700 µl of isopropanol followed by inversion and centrifugation for 15 min at 10,000*g*. The supernatant was carefully removed by pipette and pellets were washed with 1 ml of 80% (v/v) ethanol. Residual ethanol was removed by air drying 10 min at 37 °C. This was followed by resuspension of the pellet in 100 µl of water overnight at 4 °C without agitation of any kind.

DNA was then size selected with a modified SPRI bead protocol as described in ref. ^21^, with minor modifications: beads were added at 0.9×, and eluted DNA was resuspended in 50 µl of water. The concentration, purity and fragment size distribution of extracted DNA was then quantified with the Qubit fluorometer (Thermo Fisher Scientific), Nanodrop (Thermo Fisher Scientific) and TapeStation 2200 or 4200 (Agilent Technologies), respectively (Supplementary Table 1). All steps were carried out at room temperature unless otherwise stated.

### Defined mixture DNA extraction

For the defined bacterial mixture, two aliquots of lyophilized cells were obtained (item MSA-2006, ATCC) and resuspended in 500 µl of PBS. With 2.4 × 10^8^ cells provided, this gives a total theoretical mass of available DNA of approximately 2.2 µg before size selection, with 1 µg of size-selected HMW DNA required for long-read library preparation. Due to the extremely limited DNA available, as well as the lower purification requirements of this sample compared to stool, the HMW DNA extraction protocol was carried out as described above omitting the digestion and Genomic-tip purification steps. Samples were then quantified as above.

### Sequencing

Extracted DNA samples were prepared for long-read sequencing with the Oxford Nanopore Technologies (ONT) Ligation library preparation kit according to the manufacturer’s standard protocol with the addition of continuous gentle mixing during the ligation incubation step. Stool libraries were sequenced with the ONT MinION sequencer using rev C R9.4 flow cells, allocating one flowcell per sample. The defined bacterial mixture and each of the ten healthy adult samples was allocated one revolution of a D R9.4 flowcell. The sequencer was controlled with the MinKNOW v.2.2.12 software running on a MacBook Pro (model A1502, Apple), with data stored to a Vectotech 2Tb solid-state hard drive. Sequencing runs were scheduled for 48–60 h, and allowed to run until fewer than ten pores remained functional. After sequencing, data were uploaded to the Stanford Center for Genomics computational cluster for analysis. Stool sample short-read libraries were prepared and sequenced as described previously^5^. The 250-bp paired-end ATCC mixture short-read data were supplied by OneCodex. The PacBio long-read library was prepared and sequenced with one SMRT cell on a Sequel sequencing instrument (Pacific Biosciences) according to the manufacturer’s standard protocol by the University of California Davis Genome DNA Technologies Core.

### Sequence assembly

Lathe generates raw basecalled data using Guppy v.2.3.5 and produces two subassemblies in two separate runs with either Flye v.2.4.2 with the -meta parameter, or Canu v.1.8 using the -nanopore preset parameter^22^. In either case, the two runs differ by the estimated genomeSize parameter, provided as 50 and 100 m for Canu, or 100 and 250 m for Flye. The two separate assemblies are then merged with quickmerge v.0.40 (ref. ^23^) with parameters -lm 40000 -c 5 -hco 10, polished with either Racon v.1.3.2 (ref. ^24^) and Medaka v.0.6.1 (ref. ^25^) or a parallelized version of Pilon v.1.22 (ref. ^26^) for long- or short-read consensus refinement, respectively, then circularized. To parallelize Pilon, necessary for application to metagenomic assemblies, reference sequences are divided into 100-kb segments, short reads aligned to each segment downsampled to 50× coverage depth and Pilon is then used to detect errors within the reference and read subset. These errors are then aggregated across all subset runs and used to generate a refined consensus with bcftools v.1.9–107 (ref. ^27^). Errors found in homopolymers were identified with an in-house script. Sequences were then binned and annotated as previously described^5^.

Lathe applies two methods to evaluate circularity and precisely locate the genome wrap-around point in single-contig genomes, which we term circularization. The first method detects over-circularized contigs, which are those genome contigs assembled beyond the wrap-around point of the circular chromosome resulting in redundant sequences at the contig termini. This is done by self-alignment with nucmer v.3.1 (ref. ^28^) followed by analysis by a custom script. The second attempts to assemble a contig spanning across the two ends of a candidate genome. This is done by collecting reads aligning to the termini of the candidate genome, assembling with Canu, then aligning the resultant spanning contig to the candidate genome and testing for alignment consistent with a closed circular genome. The last method is conceptually similar to an existing approach^29^, but differs primarily in its parallelized implementation of spanning contig assembly and detection, which achieves a large reduction in runtime.

To detect misassemblies, Lathe searches for locations in the assembly spanned across by one or zero long reads, indicating either a total lack of support for true contiguity or support from only a single possibly chimeric read. It does this by breaking the genome into windows smaller than the average read length, then measuring coverage within each window from reads spanning the entire window. With no misassembly, an assembly produced from a given readset will have all windows spanned by the assembled reads. A misassembly within a given window will cause read alignments to be soft-clipped at the misassembly breakpoint, preventing read alignments from spanning across the breakpoint and therefore the window. Contigs are then broken at identified misassembled sites before final output generation.

Lathe was compared to the long-read assemblers miniasm v.0.2 (ref. ^30^) Ra v.0.2.1 (ref. ^31^), wtdbg2 v.2.2 (ref. ^32^) and Flye v.2.4.2 (ref. ^33^), as well as the hybrid assemblers OPERA-MS^8^ and hybridSPAdes v.3.13.0 (ref. ^34^), for the two healthy human stool (samples P1 and P2-A) (Supplementary Table 4). For hybrid approaches, we supplemented the long-read datasets with the short-read datasets previously generated for these samples^5^. Default parameters were used for all assembly approaches. Assembly N50, total size and longest contig were calculated with Quast v.5.0.0 (ref. ^11^). Lathe can be found at https://github.com/bhattlab/lathe/.

### Genome analysis

Binning was performed and evaluated as previously described^5^. Genomes were compared to reference sequences by alignment with Mummer^28^. Long and short reads were taxonomically classified with Kraken^35^, and Shannon diversity was calculated with vegan^36^. We note that classifiers developed for short reads of uniform length do not correct for the variable read length of long reads, counting relative read counts and not relative number of bases sequenced, which may slightly bias relative abundance results. rRNA presence was determined with Barrnap v.0.9 (ref. ^37^). Gene count and insertion sequence transposase count was determined with Prokka v.1.13.3 (ref. ^16^). For *P. copri*, additional insertion sequence locations were determined by mapping previously identified *P. copri* insertion sequences^5^ to the circular genome. Putative phage regions were identified with PHASTER^38^. Figures were generated with ggplot2 v.3.2.1 (ref. ^39^). Downstream analysis workflows can be found at https://github.com/bhattlab/metagenomics_workflows/.

### Novel species identification

Classifications for the unknown genome assembled in sample P1 and shown in Fig. 2 and Table 1 as *F. prausnitzii* were attempted with BLAST v.2.9.0 (ref. ^40^) against the NCBI Genbank database^41^, 16S identification with Barrnap v.0.9 (ref. ^37^) and BLAST against the Ribosomal Database Project database^42^ and NCBI 16S Archaeal and Bacterial database, and Kraken2 classification^35^. Genome sequences were compared to the assembled genome draft by alignment and post-processing with mummer^28^.

### Reporting Summary

Further information on research design is available in the Nature Research Reporting Summary linked to this article.

## Online content

Any methods, additional references, Nature Research reporting summaries, source data, extended data, supplementary information, acknowledgements, peer review information; details of author contributions and competing interests; and statements of data and code availability are available at 10.1038/s41587-020-0422-6.

## Integrated supplementary information

Supplementary Figure 1 Overview of the molecular and informatic workflow steps. Extraction consists of enzymatic degradation of bacterial cell walls followed by an initial DNA extraction in phenol-chloroform. This is followed by a proteinase K and RNase A digestion at high temperature and purification with a gravity column. Finally, small fragments are removed by modified SPRI bead size selection. After sequencing and basecalling, read sequences are assembled twice with varying genomeSize parameter values. These two assemblies are screened for sites not spanned by multiple long reads indicating misassembly, merged, and then circular sequences are identified and trimmed. The consensus sequence is refined by either short-read or long-read polishing, and final assemblies are screened once more for any misassembled sites not spanned by long reads.

Supplementary Figure 2 TapeStation traces of a variety of stool samples. Left: TapeStation traces of high molecular weight DNA extracted from ATCC MSA-2006 defined bacterial mixture and mouse stool. The curve demonstrates a high quantity (as measured by fluorescence units on the y-axis) in the >4000 bp regime for the extracted DNA. The peak at 100 bp represents the molecular weight marker standard. Right: TapeStation trace of high molecular weight DNA extracted from canine (blue), human stool sample not included in this study (green), healthy human P1 stool sample (red), healthy human sample P2-B stool sample (light brown), and healthy human P2-A stool sample (purple). The peak at 100 bp represents the molecular weight marker standard. Extractions were performed once per sample.

Supplementary Figure 3 Read length distributions versus total bases for all samples. Histograms of total bases versus read length for the 13 stool samples, sequenced with the current approach, the PacBio library, and the ATCC bacterial mixture. Read lengths vary between <1 kbp to >100 kbp, with N50 values between 5 kbp and 10 kbp.

Supplementary Figure 4 Relative abundance of organisms across approaches. Relative abundance of organisms in samples P1 and P2-A across long read, read cloud and short read libraries, stratified by Gram stain characteristics. A chief concern of bacterial lysis methods is systematic taxonomic bias, particularly with regard to cell wall structure. Although precise rank order abundances are not identical between long and short read based approaches, deviations do not assort with broad taxonomic differences in cell wall structure in the two stool samples.

Supplementary Figure 5 Read length distributions per organism in long read sequencing from human stool samples. Although small variations between organisms are visible, overall read length distributions are visibly more consistent in stool DNA extractions than the defined bacterial mixture. *E. coli* demonstrates a visible peak in read length distribution corresponding to reads originating from conserved sequences most likely misattributed to these organisms (see text).

Supplementary Figure 6 Bin counts for nanopore, read cloud and short read approaches. (**a**) High quality genome bins with a minimum N50. (**b**) High quality genome bins below a given depth of read coverage. (**c**) High quality genome bins with an N50 exceeding 2 Mbp below a given read coverage depth.

Supplementary Figure 7 Reference alignment dotplots for closed genomes obtained by nanopore long read sequencing and assembly. Although assemblies share broad structural similarity to available references, there are cases where observed organisms are significantly structurally diverged (for example *Dialister*) and in one case bears minimal similarity to the closest available reference (*Faecalibacterium*; note shorter *x*-axis). Asterisks denotes genome later annotated as putative *Cibiobacter*.

Supplementary Figure 8 TapeStation quantification of DNA fragments obtained from healthy adult samples. (**a**) TapeStation quantification of DNA extracted from healthy adult stool samples A (green), C (blue), and E (yellow), prior to size selection. (**b**) TapeStation quantification of samples from panel A, after size selection with SPRI beads. All but one sample (A, green) yielded very short fragments and insufficient DNA after size selection. (**c**) TapeStation quantification of DNA extracted from eight healthy adult stool samples (A, B, C, E, F, G, H, J) after extraction with the present approach shows a significant enrichment for DNA fragments above 10 kb and minimal shorter fragments. Extractions were performed once per sample.

Supplementary Figure 9 Taxonomic composition across extraction and sequencing methods. Taxonomic composition of healthy adult stool sample A, which was subjected to bead beating followed by nanopore sequencing vs. short read sequencing; and which was also subjected to the high molecular weight extraction and nanopore sequencing. Only one of the ten additional healthy adult stool samples that were bead beaten yielded sufficient quantities of SPRI size-selected DNA for subsequent nanopore sequencing. All other samples yielded short fragments by TapeStation quantification (Supplementary Fig. 8). The ten most abundant taxa are depicted in this figure for clarity of representation.

Supplementary Figure 10 Sequence-derived taxonomic composition of additional healthy adult cohort samples. Ten additional healthy adult stool samples were subjected to both the present approach (hmw) and a conventional approach (sr) consisting of bead-beating lysis in conjunction with short read sequencing. The eleven most abundant species in short read sequencing data are shown in both libraries for visual clarity. The organisms most highly represented in the conventional approach are recovered by the present approach in all cases.

Supplementary Figure 11 Genus-level comparison of bacterial relative abundances across extraction and sequencing approaches. **a**) Comparison of relative abundances of most abundant genera in healthy human stool samples (n=10) processed with bead beating and short read sequencing (SR) or the present approach and nanopore sequencing (HMW). Only genera with median relative abundances of >1% are shown for visual clarity. Boxes represent quartiles and median values, whiskers represent maximum and minimum values or quartiles ± 1.5 times the interquartile range, and points represent outliers. **b**) Comparison of log2 fold change of genera between samples extracted with HMW and SR approaches (n=10) demonstrates that bias for certain genera is consistent across samples. A single asterisk indicates a p-value < 0.05, two-sided Wilcoxon signed-rank test.

Supplementary Figure 12 Comparison of species-level read counts across extraction and sequencing approaches. Read counts of species detected by the gold standard approach of bead beating and short read sequencing (x-axis) versus read counts of species detected by the present approach incorporating supplemental lytic enzymes (see Methods) (y-axis). On the log-transformed read counts, the two approaches show a Pearson correlation of 0.79 across samples (n=10). In addition, of the 18,462 total cases in which a given species was more than ten-fold enriched in relative abundance in either approach over the other, we found that our approach yielded the higher relative abundance in 95% of cases, suggesting the potential for richer taxonomic sensitivity by our method.

Supplementary Figure 13 Contiguity, size and quality of species genome draft sequences obtained by the present approach from ten healthy adult stool samples. The present approach remains capable of yielding high quality (>90% completeness, <5% contamination, at least 1 each of 5 S, 16 S and 23 S rRNA, at least 18 tRNA loci), contiguous drafts when applied to additional complex samples. Drafts are shown for all organisms with at least 2% relative abundance, at least intermediate quality (high quality with minimum completeness reduced to 75%), or an N50 of at least 1 Mbp. For each species genome draft on the y-axis, the draft N50 (left) or the total draft length (right) is shown on the x-axis. If more than one draft genome per organism was generated from the same sample, only the draft with the highest N50 is shown for clarity.

Supplementary Figure 14 Limitations of long read assembly. **a**) Assembly graphs demonstrating uniquely assemblable sequences present in long read data. The left sequence belongs to contigs comprising the assembled genome of *Prevotella copri*, which is distinct enough from other organisms in the community to prevent ambiguous paths through multiple genomes. The right sequence belongs to a complex of *Bacteroides* genomes within which the genome of *Bacteroides vulgatus* is indicated in colored strands. This complex arises from a higher number of genomically similar organisms in admixture within the community. This creates a high number of ambiguous junctions in the assembly graph where multiple unique sequences can be assembled, visible as loops in this visualization. Long reads disambiguate these junctions when they are sufficiently long and well-positioned to reveal true paths through the graph, and the odds of this occurring are increased with higher raw read N50. **b**) Assembly contiguity, expressed as per-bin N50 divided by total bin length, as a function of total count of bins of the same genus and sample for bins from samples P1, P2-A, P2-B, and P2-coassembled that had >300x coverage, >1 Mbp total length, and ≤ 3 other bins from same genus (n = 24). As genome contiguity approaches completion, the value N50 divided by total length approaches one. With more bins from the same genus within a given community, the observed bin assembly contiguity is reduced. This is attributable to the increased likelihood of highly similar sequences occurring in multiple genomes. Line indicates fitted generalized linear model and shading indicates 95% confidence interval.

## Data Availability

All sequence data, whole metagenome assemblies and individual completed genomes can be found at the NCBI BioProject under accession code PRJNA508395.

## Supplemental information

Supplementary Information Supplementary Figs. 1–14 and Notes 1 and 2.
Reporting Summary
Supplementary Table 1 HMW DNA statistics
Supplementary Table 2 Sequencing data yields
Supplementary Table 3 Mock community assembly and polishing comparisons
Supplementary Table 4 Mock community assembler comparisons
Supplementary Table 5 Human gut assembly summaries
Supplementary Table 6 Extraction, library preparation, and sequencing costs
Supplementary Table 7 Additional human gut binning summaries

## Code availability

Lathe is available at https://github.com/bhattlab/lathe. Version 1.0 was used for analyses in this paper. Binning, annotation and post-processing workflows can be found at https://github.com/bhattlab/metagenomics_workflows.

## References

[1] Bowers RM (2017). Minimum information about a single amplified genome (MISAG) and a metagenome-assembled genome (MIMAG) of bacteria and archaea. Nat. Biotechnol..

[2] Kang DD, Froula J, Egan R, Wang Z (2015). MetaBAT, an efficient tool for accurately reconstructing single genomes from complex microbial communities. PeerJ.

[3] Forster SC (2019). A human gut bacterial genome and culture collection for improved metagenomic analyses. Nat. Biotechnol..

[4] Nayfach S, Shi ZJ, Seshadri R, Pollard KS, Kyrpides N (2019). Novel insights from uncultivated genomes of the global human gut microbiome.. Nature.

[5] Bishara A (2018). High-quality genome sequences of uncultured microbes by assembly of read clouds.. Nat. Biotechnol..

[6] Pendleton M (2015). Assembly and diploid architecture of an individual human genome via single-molecule technologies. Nat. Methods.

[7] Stewart RD (2019). Compendium of 4,941 rumen metagenome-assembled genomes for rumen microbiome biology and enzyme discovery. Nat. Biotechnol..

[8] Bertrand D (2019). Hybrid metagenomic assembly enables high-resolution analysis of resistance determinants and mobile elements in human microbiomes. Nat. Biotechnol..

[9] Branton, D. & Deamer, D. *Nanopore Seqeuncing* (World Scientific, 2019).

[10] Bankevich A (2012). SPAdes: a new genome assembly algorithm and its applications to single-cell sequencing. J. Comput. Biol..

[11] Mikheenko A, Saveliev V, Gurevich A (2015). MetaQUAST: evaluation of metagenome assemblies. Bioinformatics.

[12] Albertsen M (2013). Genome sequences of rare, uncultured bacteria obtained by differential coverage binning of multiple metagenomes. Nat. Biotechnol..

[13] Parks DH, Imelfort M, Skennerton CT, Hugenholtz P, Tyson GW (2015). CheckM: assessing the quality of microbial genomes recovered from isolates, single cells, and metagenomes. Genome Res..

[14] Chen, L.-X., Anantharaman, K., Shaiber, A., Murat Eren, A. & Banfield, J. F. Accurate and complete genomes from metagenomes. Preprint at *bioRxiv*10.1101/808410 808410 (2019). 10.1101/gr.258640.119 PMC7111523 32188701

[15] Gupta VK, Chaudhari NM, Iskepalli S, Dutta C (2015). Divergences in gene repertoire among the reference prevotella genomes derived from distinct body sites of human. BMC Genomics.

[16] Seemann T (2014). Prokka: rapid prokaryotic genome annotation. Bioinformatics.

[17] Pasolli E (2019). Extensive unexplored human microbiome diversity revealed by over 150,000 genomes from metagenomes spanning age, geography, and lifestyle. Cell.

[18] Zeevi D (2019). Structural variation in the gut microbiome associates with host health.. Nature.

[19] De Filippis F (2019). Distinct genetic and functional traits of human intestinal *Prevotella copri* strains are associated with different habitual diets. Cell Host Microbe.

[20] Beaulaurier J (2018). Metagenomic binning and association of plasmids with bacterial host genomes using DNA methylation. Nat. Biotechnol..

[21] Nagar, R. & Schwessinger, B. DNA size selection (>3–4 kb) and purification of DNA using an improved homemade SPRI beads solution. v.1. *Protocols.io*10.17504/protocols.io.n7hdhj6 (2018).

[22] Koren S (2017). Canu: scalable and accurate long-read assembly via adaptive k-mer weighting and repeat separation.. Genome Res..

[23] Chakraborty M, Baldwin-Brown JG, Long AD, Emerson JJ (2016). Contiguous and accurate de novo assembly of metazoan genomes with modest long read coverage. Nucleic Acids Res..

[24] Vaser R, Sović I, Nagarajan N, Šikić M (2017). Fast and accurate de novo genome assembly from long uncorrected reads. Genome Res..

[25] *Medaka 0.3.0 Documentation* (Oxford Nanopore Technologies, 2018); https://nanoporetech.github.io/medaka/index.html

[26] Walker BJ (2014). Pilon: an integrated tool for comprehensive microbial variant detection and genome assembly improvement. PLoS ONE.

[27] Danecek, P. Others. bcftools—utilities for variant calling and manipulating vcfs and bcfs (GitHub, 2015).

[28] Delcher AL, Salzberg SL, Phillippy AM (2003). Using MUMmer to identify similar regions in large sequence sets. Curr. Protoc. Bioinformatics.

[29] Hunt M (2015). Circlator: automated circularization of genome assemblies using long sequencing reads. Genome Biol..

[30] Li H (2016). Minimap and miniasm: fast mapping and de novo assembly for noisy long sequences. Bioinformatics.

[31] Vaser, R. ra v0.2.1 (Github).

[32] Ruan J, Li H (2019). Fast and accurate long-read assembly with wtdbg2. Nat. Methods.

[33] Kolmogorov M, Yuan J, Lin Y, Pevzner PA (2019). Assembly of long, error-prone reads using repeat graphs.. Nat. Biotechnol..

[34] Antipov D, Korobeynikov A, McLean JS, Pevzner PA (2016). hybridSPAdes: an algorithm for hybrid assembly of short and long reads. Bioinformatics.

[35] Wood D, Salzberg S (2014). Kraken: ultrafast metagenomic sequence classification using exact alignments. Genome Biol..

[36] Dixon P (2003). VEGAN, a package of R functions for community ecology. J. Veg. Sci..

[37] Seemann, T. barrnap v2.2 (Github).

[38] Arndt D (2016). PHASTER: a better, faster version of the PHAST phage search tool. Nucleic Acids Res..

[39] Wickham, H. *ggplot2: Elegant Graphics for Data Analysis* (Springer Science & Business Media, 2009).

[40] Altschul SF, Gish W, Miller W, Myers EW, Lipman DJ (1990). Basic local alignment search tool. J. Mol. Biol..

[41] Benson DA (2013). GenBank. Nucleic Acids Res..

[42] Maidak BL (1997). The RDP (Ribosomal Database Project).. Nucleic Acids Res..

