# Supplementary figures and images for: Complete, closed bacterial genomes from microbiomes using nanopore sequencing
Source: Nat Biotechnol. 2020 Feb 10;38(6):701–7. doi: 10.1038/s41587-020-0422-6 (PMC7283042; doi:10.1038/s41587-020-0422-6)

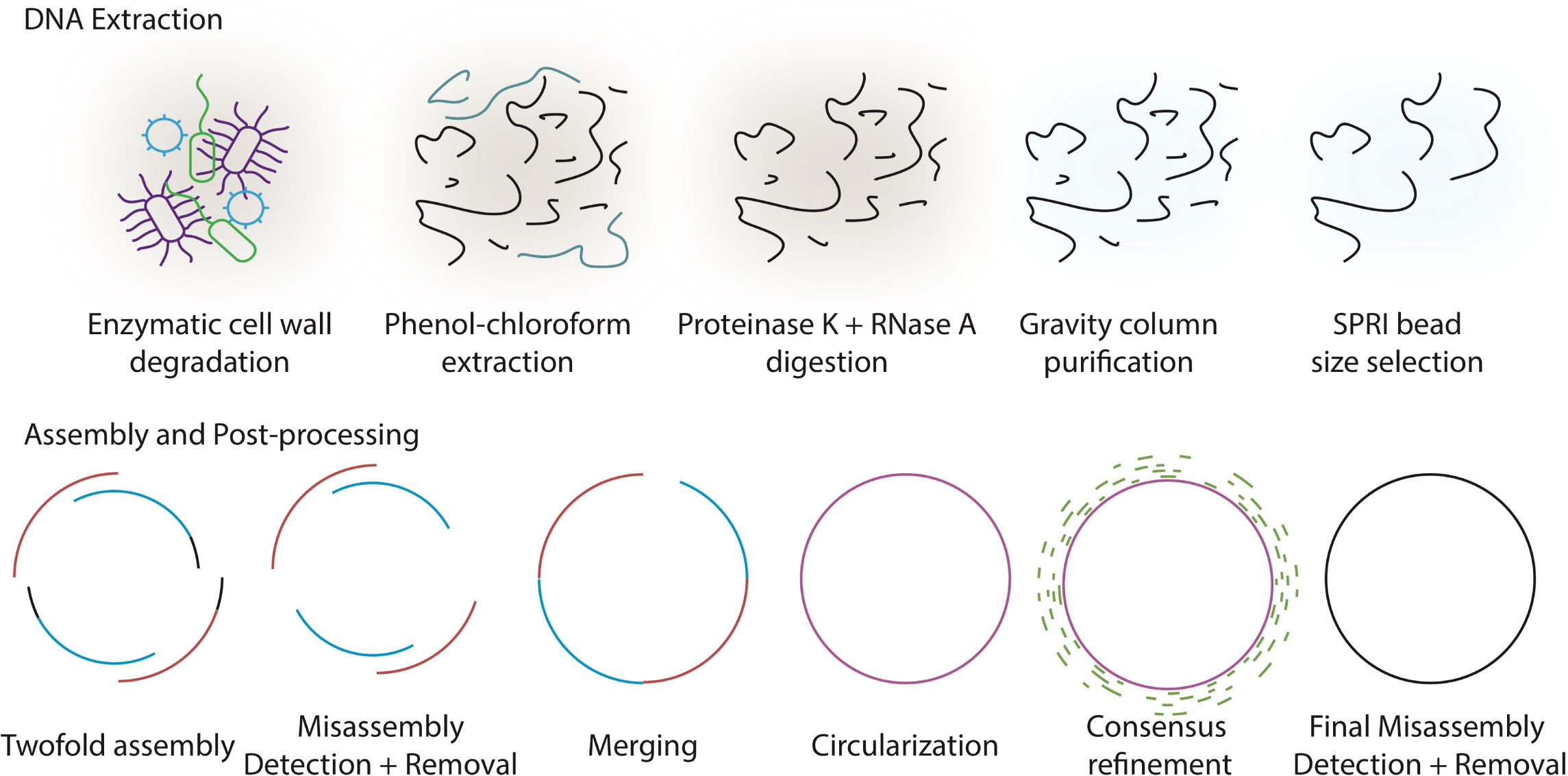

Supplement: Overview of the molecular and informatic workflow steps. — Extraction consists of enzymatic degradation of bacterial cell walls followed by an initial DNA extraction in phenol-chloroform. This is followed by a proteinase K and RNase A digestion at high temperature and purification with a gravity column. Finally, small fragments are removed by modified SPRI bead size selection. After sequencing and basecalling, read sequences are assembled twice with varying genomeSize parameter values. These two assemblies are screened for sites not spanned by multiple long reads indicating misassembly, merged, and then circular sequences are identified and trimmed. The consensus sequence is refined by either short-read or long-read polishing, and final assemblies are screened once more for any misassembled sites not spanned by long reads. [file 41587_2020_422_Fig4_ESM.jpg]

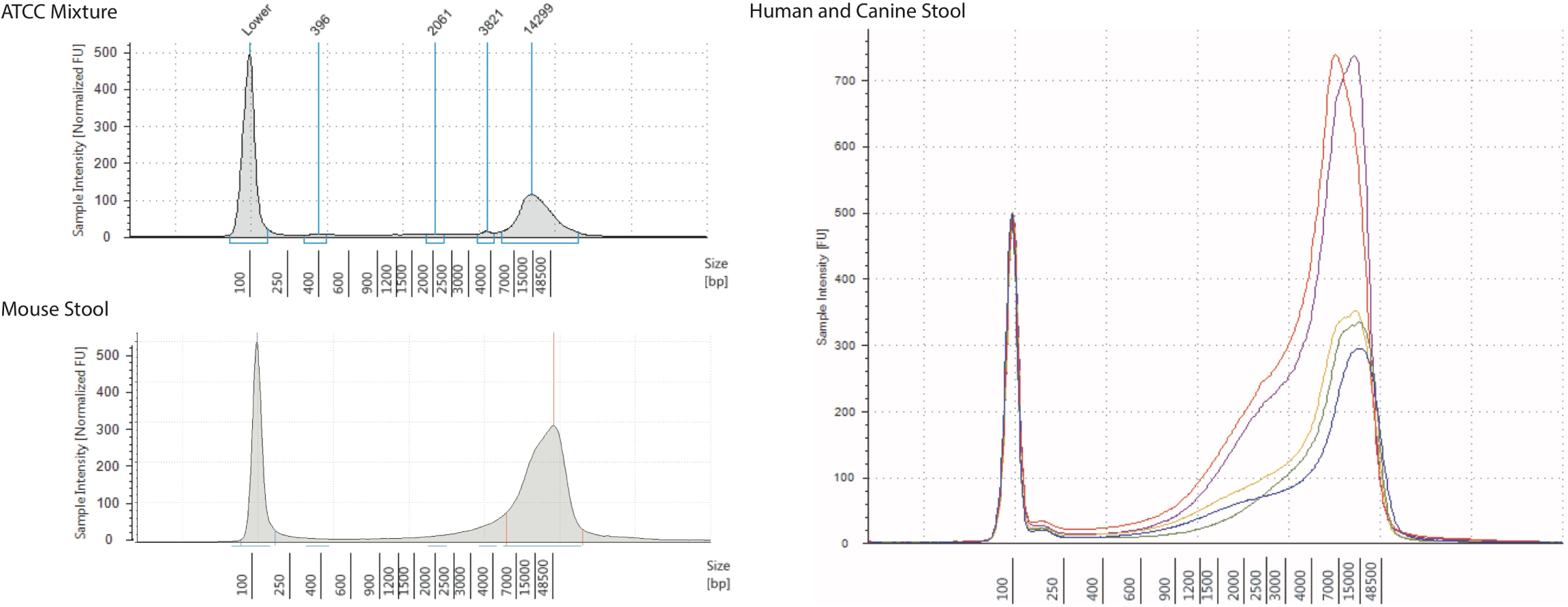

Supplement: TapeStation traces of a variety of stool samples. — Left: TapeStation traces of high molecular weight DNA extracted from ATCC MSA-2006 defined bacterial mixture and mouse stool. The curve demonstrates a high quantity (as measured by fluorescence units on the y-axis) in the >4000 bp regime for the extracted DNA. The peak at 100 bp represents the molecular weight marker standard. Right: TapeStation trace of high molecular weight DNA extracted from canine (blue), human stool sample not included in this study (green), healthy human P1 stool sample (red), healthy human sample P2-B stool sample (light brown), and healthy human P2-A stool sample (purple). The peak at 100 bp represents the molecular weight marker standard. Extractions were performed once per sample. [file 41587_2020_422_Fig5_ESM.jpg]

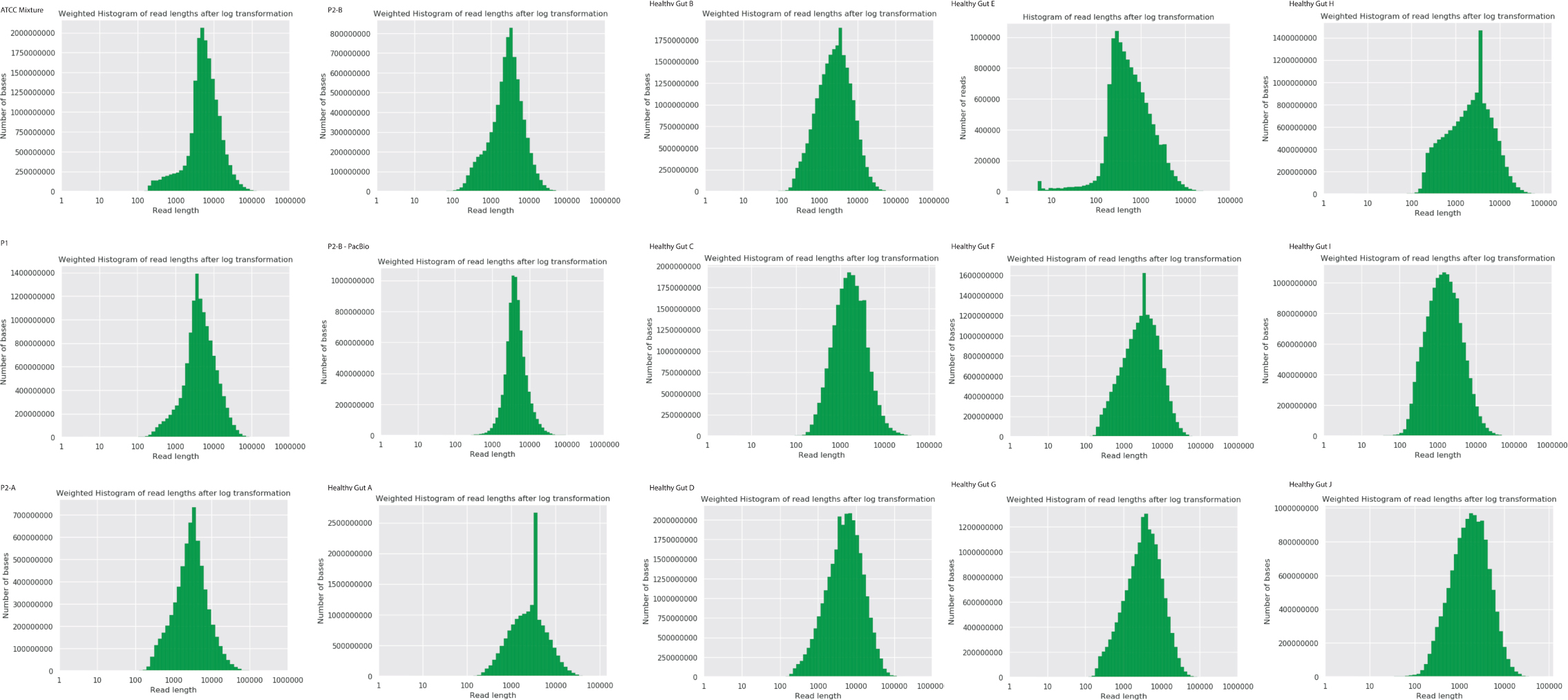

Supplement: Read length distributions versus total bases for all samples. — Histograms of total bases versus read length for the 13 stool samples, sequenced with the current approach, the PacBio library, and the ATCC bacterial mixture. Read lengths vary between <1 kbp to >100 kbp, with N50 values between 5 kbp and 10 kbp. [file 41587_2020_422_Fig6_ESM.jpg]

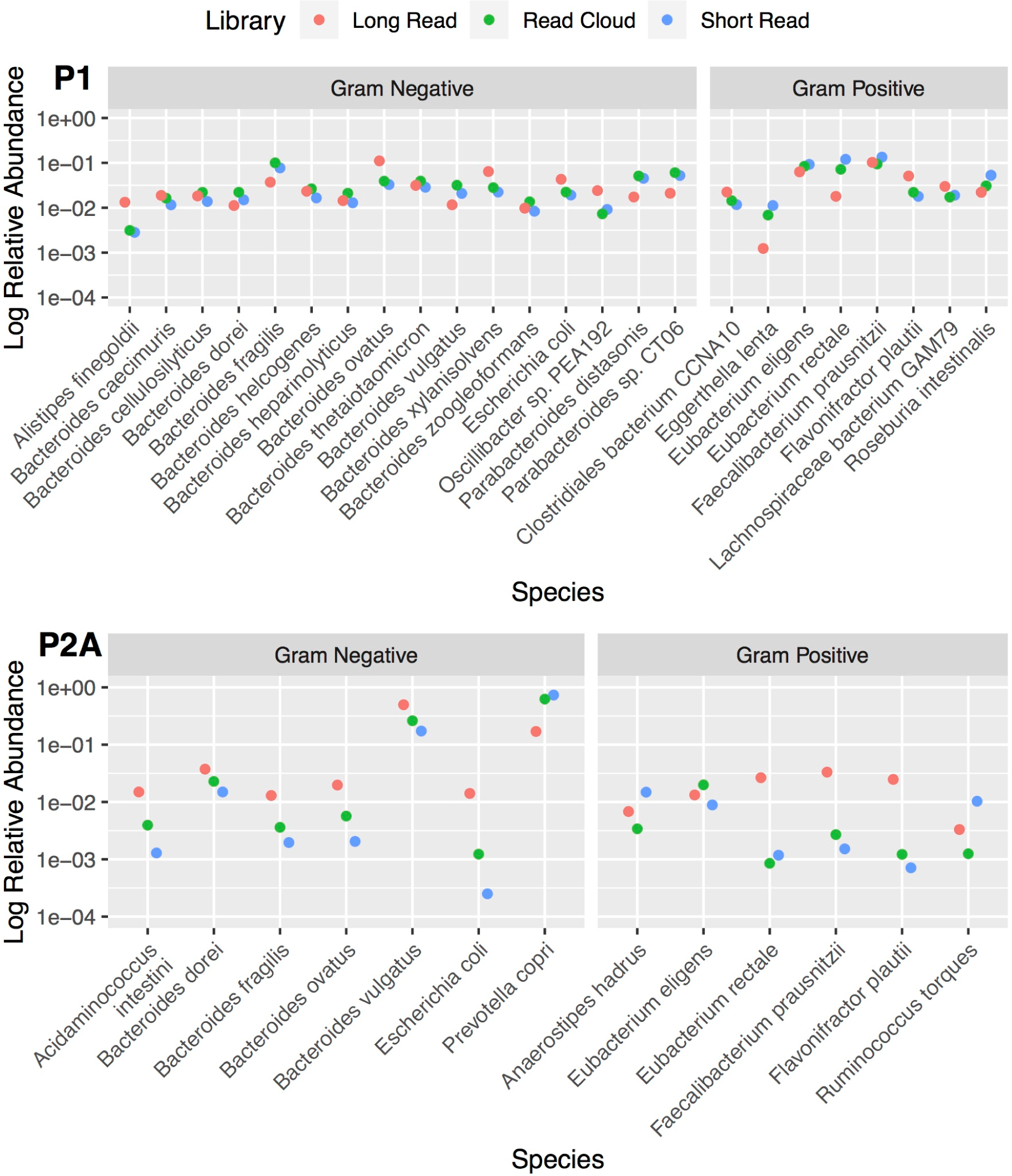

Supplement: Relative abundance of organisms across approaches. — Relative abundance of organisms in samples P1 and P2-A across long read, read cloud and short read libraries, stratified by Gram stain characteristics. A chief concern of bacterial lysis methods is systematic taxonomic bias, particularly with regard to cell wall structure. Although precise rank order abundances are not identical between long and short read based approaches, deviations do not assort with broad taxonomic differences in cell wall structure in the two stool samples. [file 41587_2020_422_Fig7_ESM.jpg]

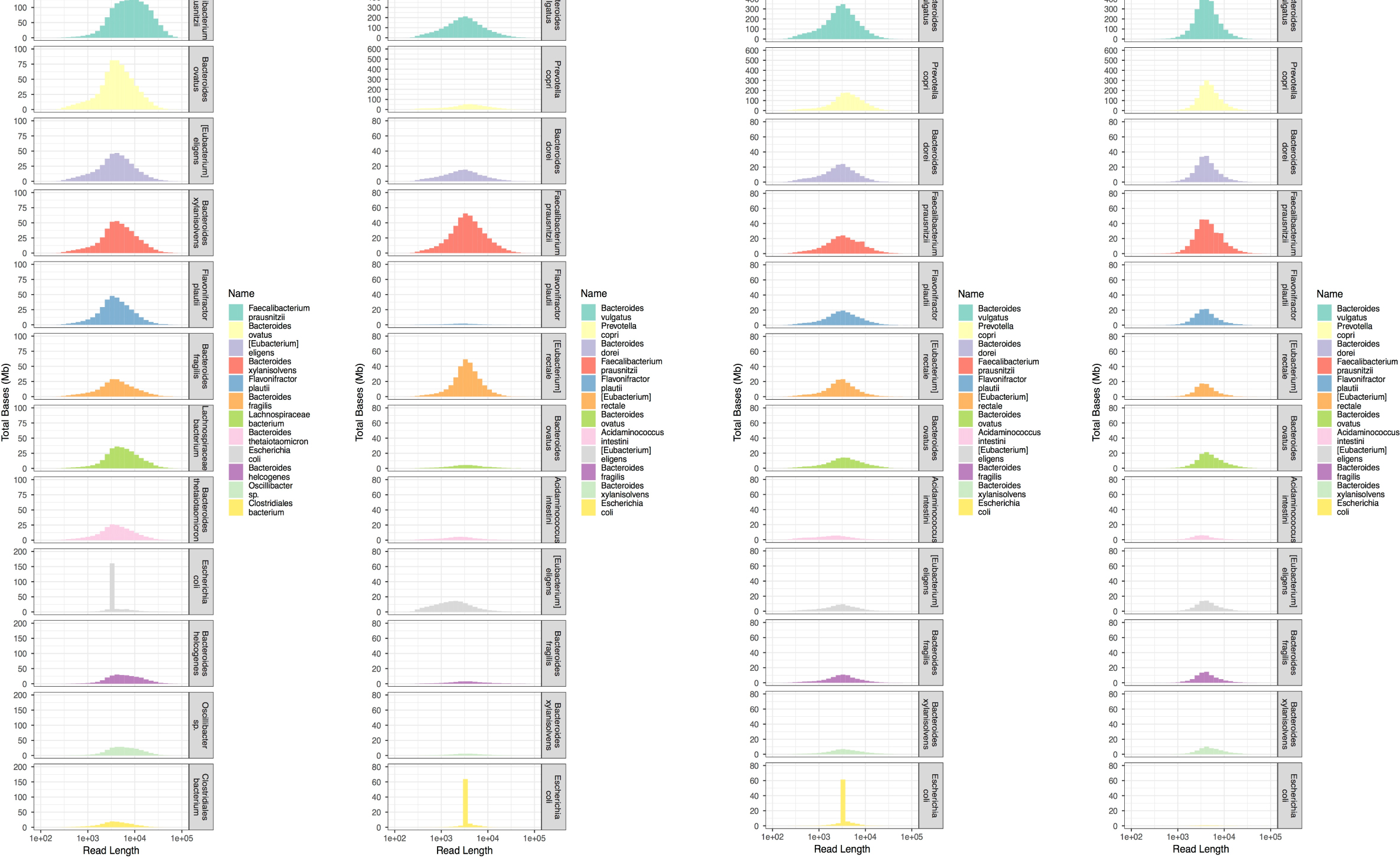

Supplement: Read length distributions per organism in long read sequencing from human stool samples. — Although small variations between organisms are visible, overall read length distributions are visibly more consistent in stool DNA extractions than the defined bacterial mixture. E. coli demonstrates a visible peak in read length distribution corresponding to reads originating from conserved sequences most likely misattributed to these organisms (see text). [file 41587_2020_422_Fig8_ESM.jpg]

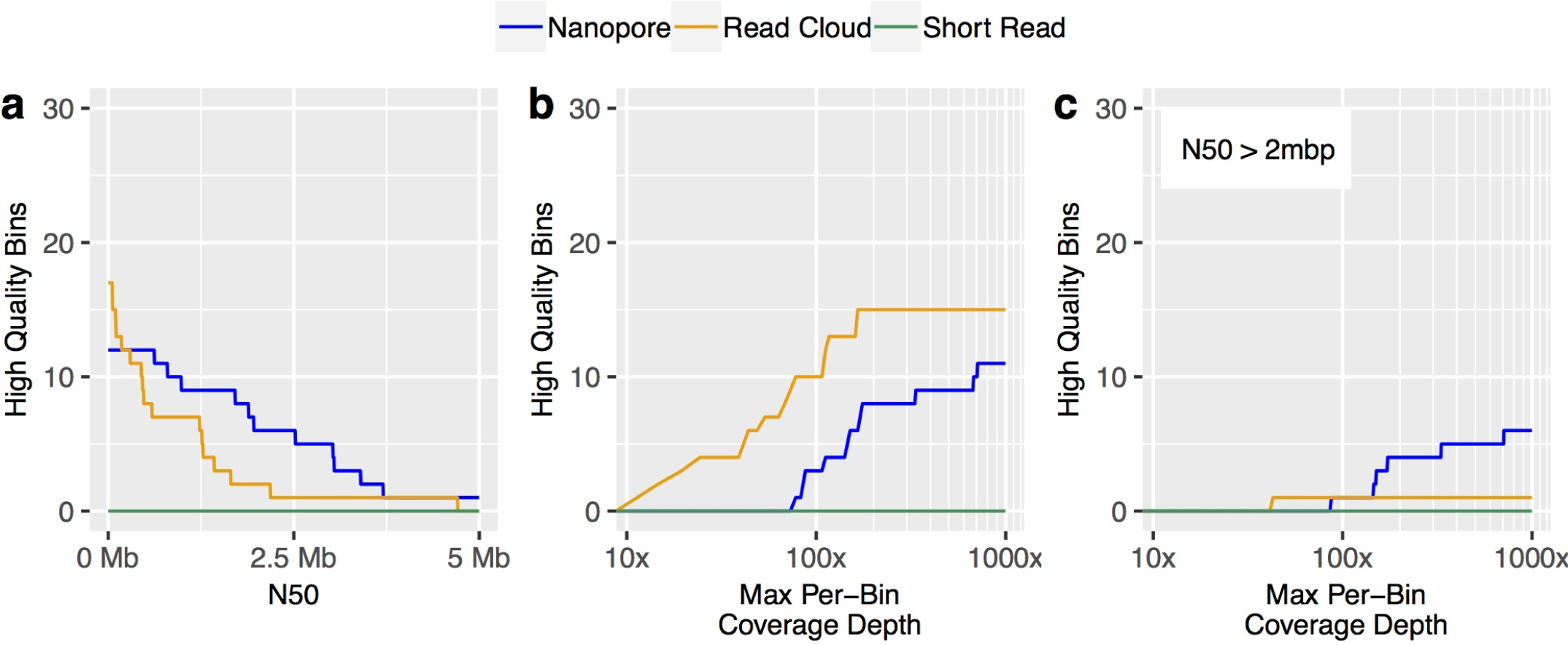

Supplement: Bin counts for nanopore, read cloud and short read approaches. — (a) High quality genome bins with a minimum N50. (b) High quality genome bins below a given depth of read coverage. (c) High quality genome bins with an N50 exceeding 2 Mbp below a given read coverage depth. [file 41587_2020_422_Fig9_ESM.jpg]

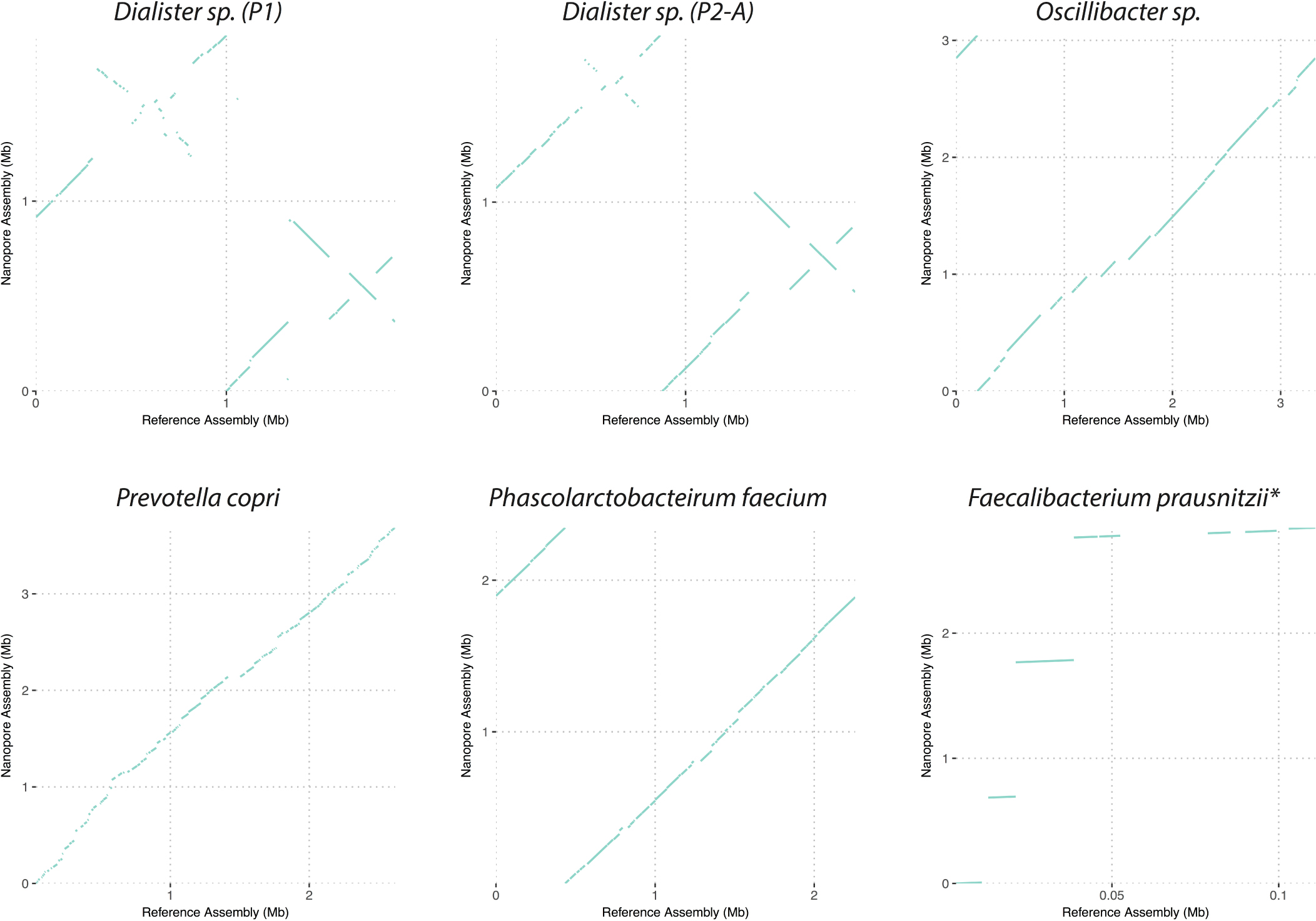

Supplement: Reference alignment dotplots for closed genomes obtained by nanopore long read sequencing and assembly. — Although assemblies share broad structural similarity to available references, there are cases where observed organisms are significantly structurally diverged (for example Dialister) and in one case bears minimal similarity to the closest available reference (Faecalibacterium; note shorter x-axis). Asterisks denotes genome later annotated as putative Cibiobacter. [file 41587_2020_422_Fig10_ESM.jpg]

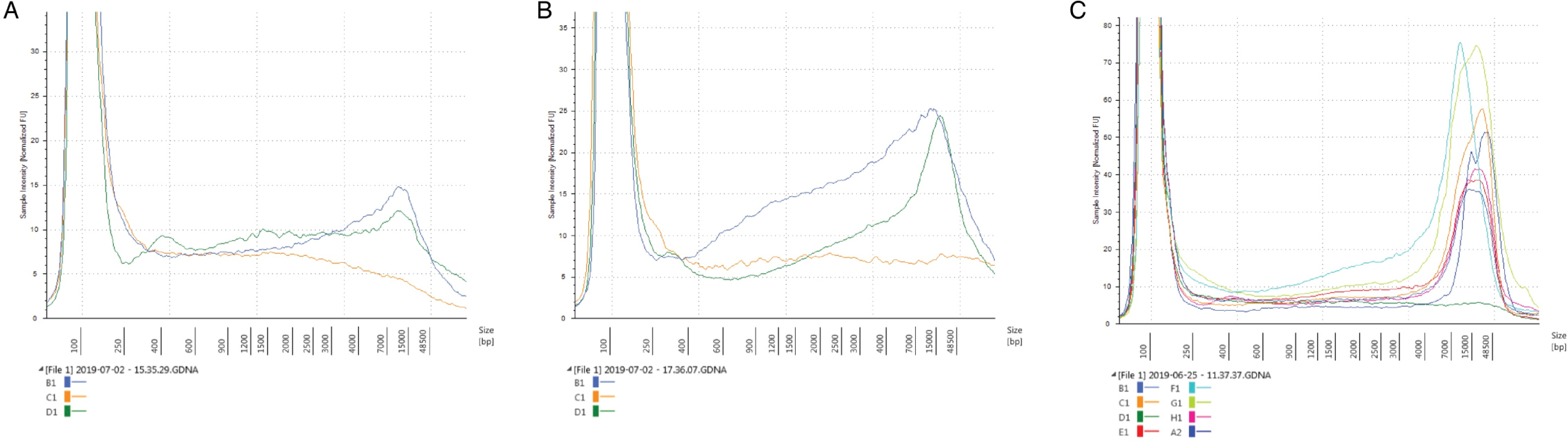

Supplement: TapeStation quantification of DNA fragments obtained from healthy adult samples. — (a) TapeStation quantification of DNA extracted from healthy adult stool samples A (green), C (blue), and E (yellow), prior to size selection. (b) TapeStation quantification of samples from panel A, after size selection with SPRI beads. All but one sample (A, green) yielded very short fragments and insufficient DNA after size selection. (c) TapeStation quantification of DNA extracted from eight healthy adult stool samples (A, B, C, E, F, G, H, J) after extraction with the present approach shows a significant enrichment for DNA fragments above 10 kb and minimal shorter fragments. Extractions were performed once per sample. [file 41587_2020_422_Fig11_ESM.jpg]

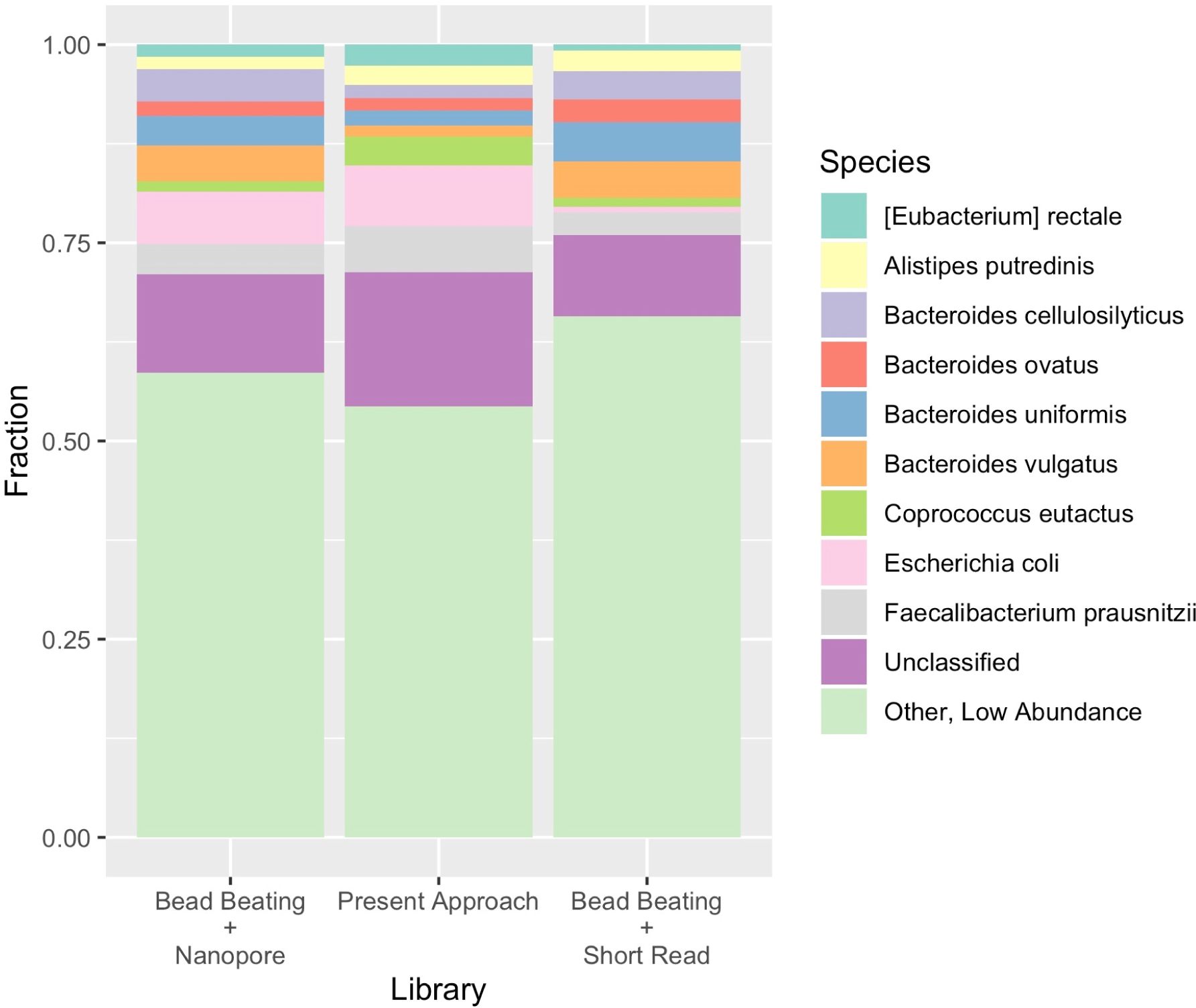

Supplement: Taxonomic composition across extraction and sequencing methods. — Taxonomic composition of healthy adult stool sample A, which was subjected to bead beating followed by nanopore sequencing vs. short read sequencing; and which was also subjected to the high molecular weight extraction and nanopore sequencing. Only one of the ten additional healthy adult stool samples that were bead beaten yielded sufficient quantities of SPRI size-selected DNA for subsequent nanopore sequencing. All other samples yielded short fragments by TapeStation quantification (Supplementary Fig. 8). The ten most abundant taxa are depicted in this figure for clarity of representation. [file 41587_2020_422_Fig12_ESM.jpg]

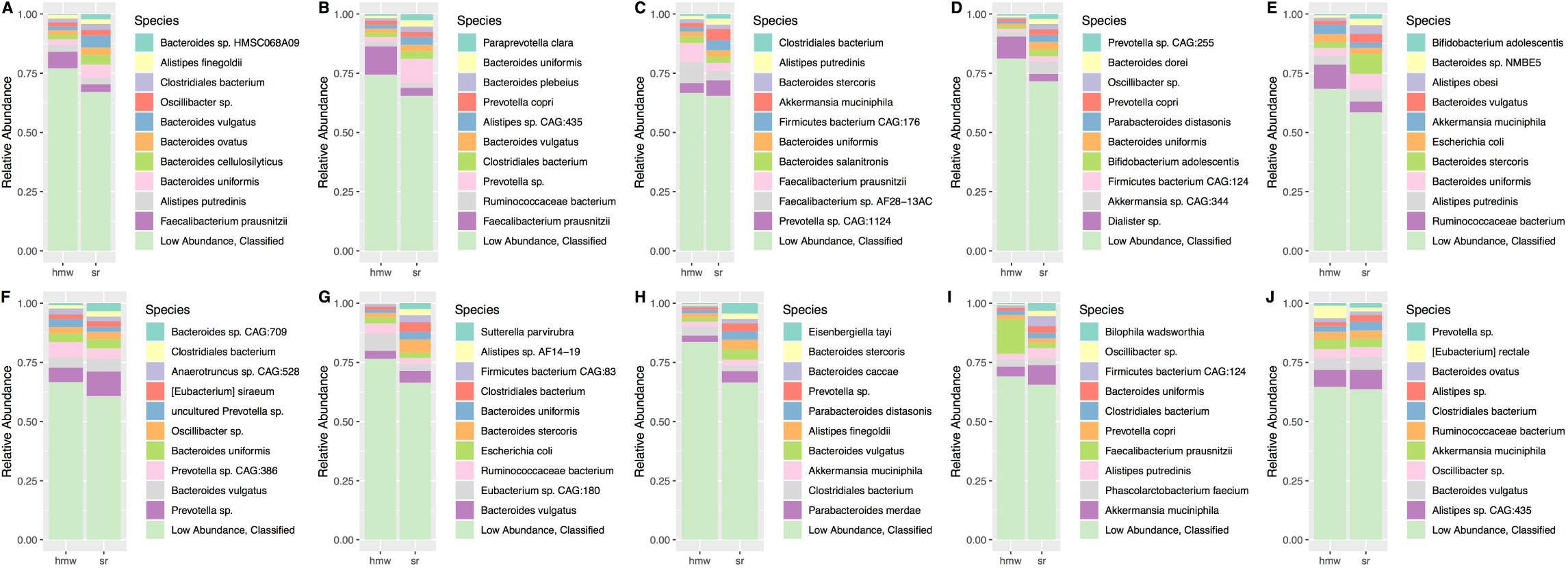

Supplement: Sequence-derived taxonomic composition of additional healthy adult cohort samples. — Ten additional healthy adult stool samples were subjected to both the present approach (hmw) and a conventional approach (sr) consisting of bead-beating lysis in conjunction with short read sequencing. The eleven most abundant species in short read sequencing data are shown in both libraries for visual clarity. The organisms most highly represented in the conventional approach are recovered by the present approach in all cases. [file 41587_2020_422_Fig13_ESM.jpg]

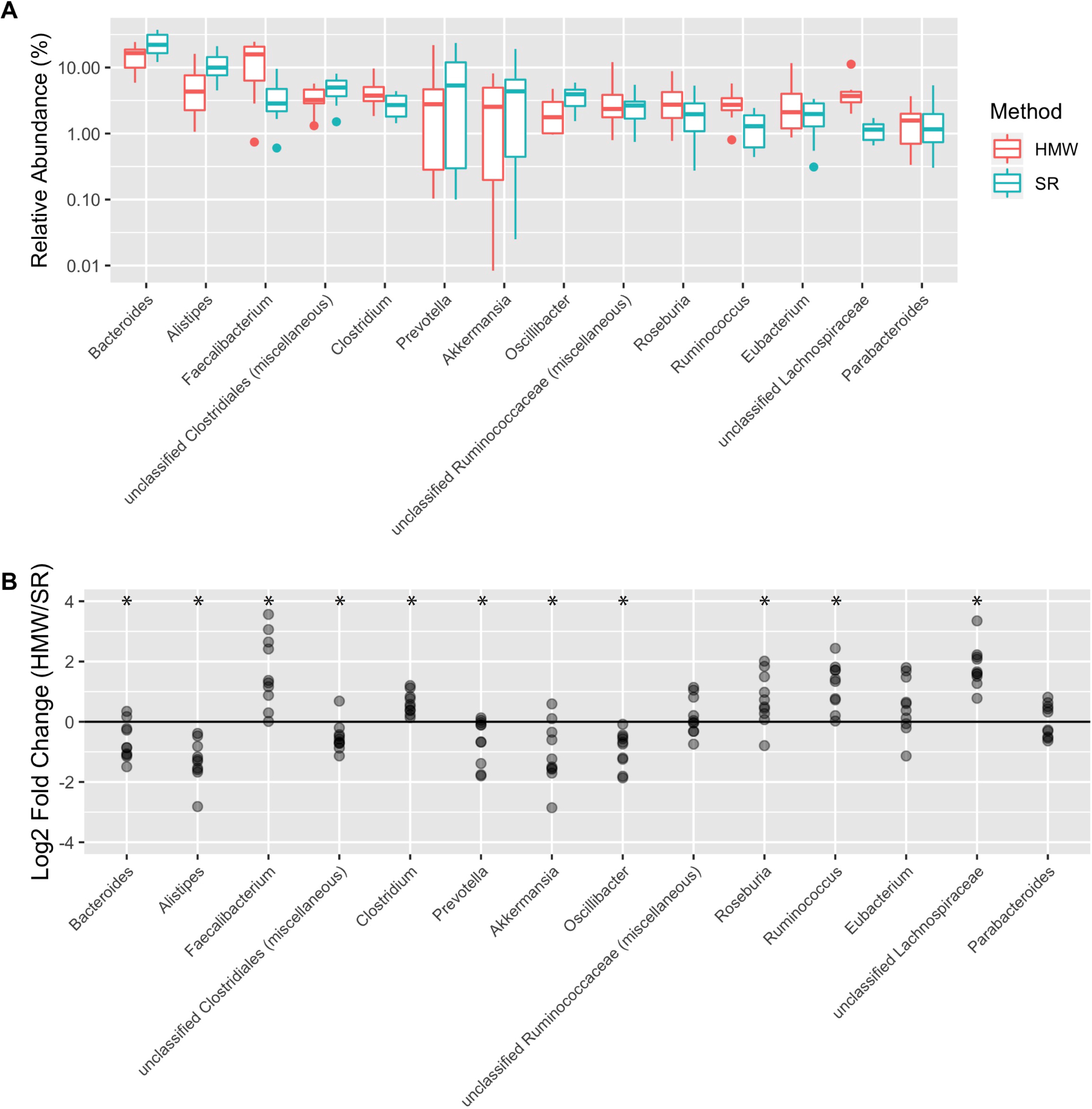

Supplement: Genus-level comparison of bacterial relative abundances across extraction and sequencing approaches. — a) Comparison of relative abundances of most abundant genera in healthy human stool samples (n=10) processed with bead beating and short read sequencing (SR) or the present approach and nanopore sequencing (HMW). Only genera with median relative abundances of >1% are shown for visual clarity. Boxes represent quartiles and median values, whiskers represent maximum and minimum values or quartiles ± 1.5 times the interquartile range, and points represent outliers. b) Comparison of log2 fold change of genera between samples extracted with HMW and SR approaches (n=10) demonstrates that bias for certain genera is consistent across samples. A single asterisk indicates a p-value < 0.05, two-sided Wilcoxon signed-rank test. [file 41587_2020_422_Fig14_ESM.jpg]

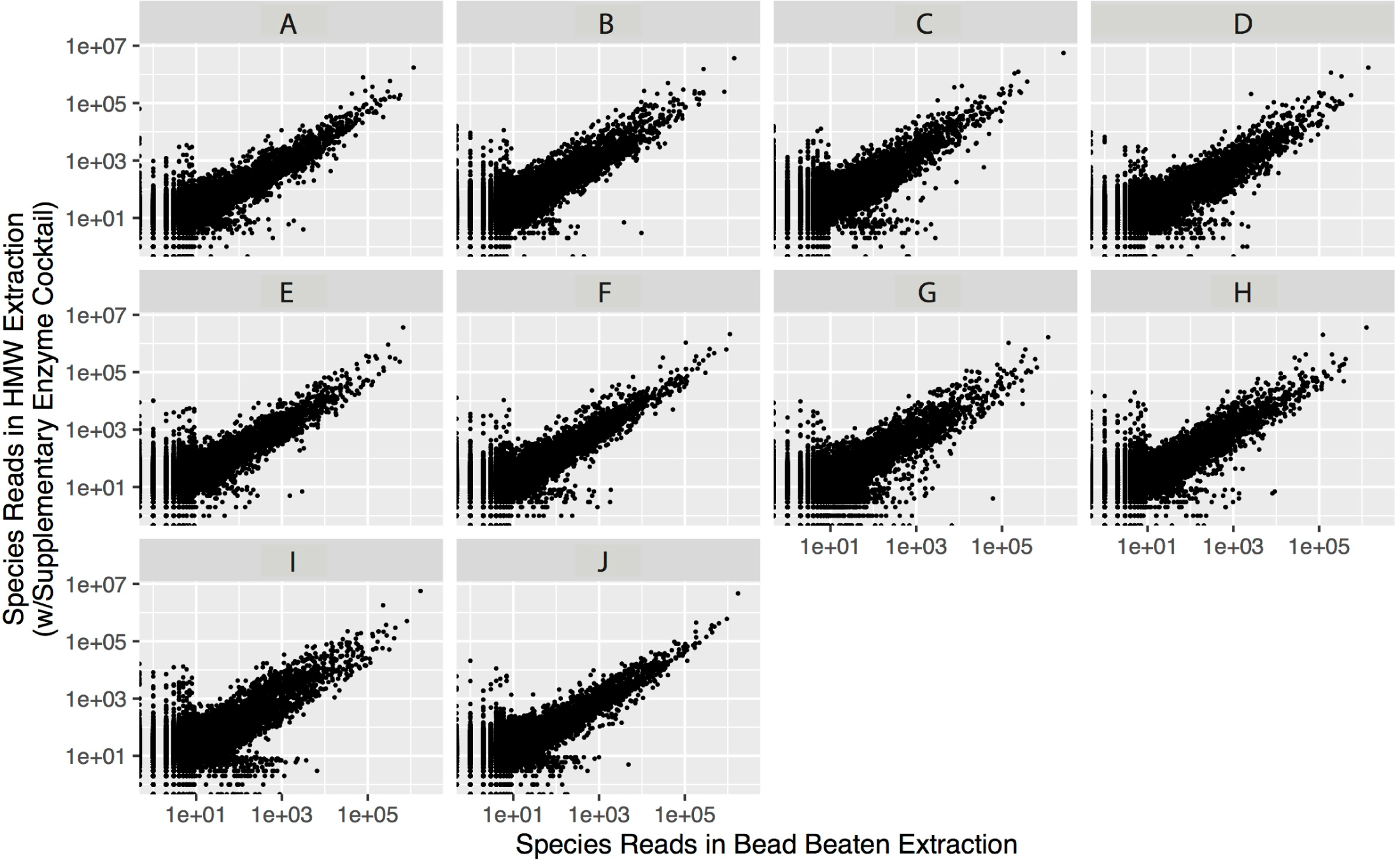

Supplement: Comparison of species-level read counts across extraction and sequencing approaches. — Read counts of species detected by the gold standard approach of bead beating and short read sequencing (x-axis) versus read counts of species detected by the present approach incorporating supplemental lytic enzymes (see Methods) (y-axis). On the log-transformed read counts, the two approaches show a Pearson correlation of 0.79 across samples (n=10). In addition, of the 18,462 total cases in which a given species was more than ten-fold enriched in relative abundance in either approach over the other, we found that our approach yielded the higher relative abundance in 95% of cases, suggesting the potential for richer taxonomic sensitivity by our method. [file 41587_2020_422_Fig15_ESM.jpg]

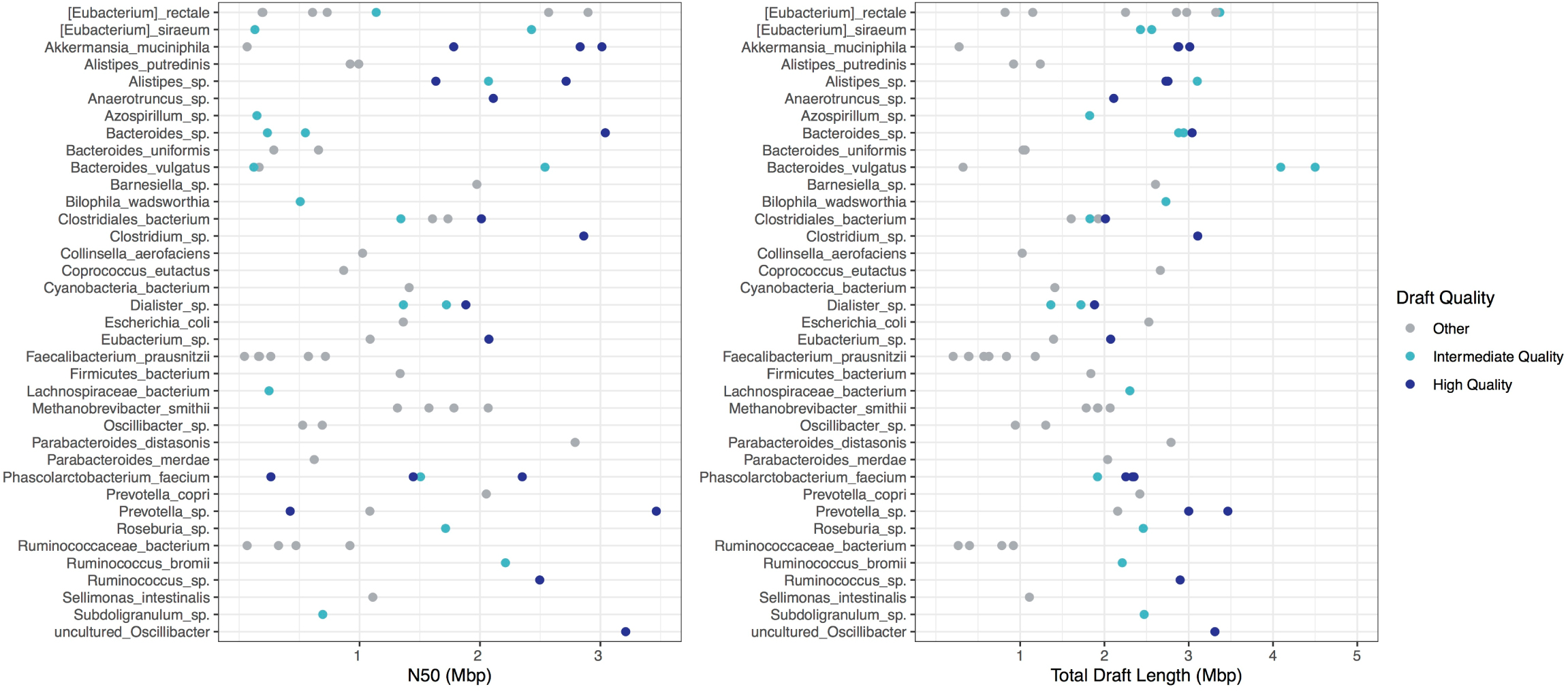

Supplement: Contiguity, size and quality of species genome draft sequences obtained by the present approach from ten healthy adult stool samples. — The present approach remains capable of yielding high quality (>90% completeness, <5% contamination, at least 1 each of 5 S, 16 S and 23 S rRNA, at least 18 tRNA loci), contiguous drafts when applied to additional complex samples. Drafts are shown for all organisms with at least 2% relative abundance, at least intermediate quality (high quality with minimum completeness reduced to 75%), or an N50 of at least 1 Mbp. For each species genome draft on the y-axis, the draft N50 (left) or the total draft length (right) is shown on the x-axis. If more than one draft genome per organism was generated from the same sample, only the draft with the highest N50 is shown for clarity. [file 41587_2020_422_Fig16_ESM.jpg]

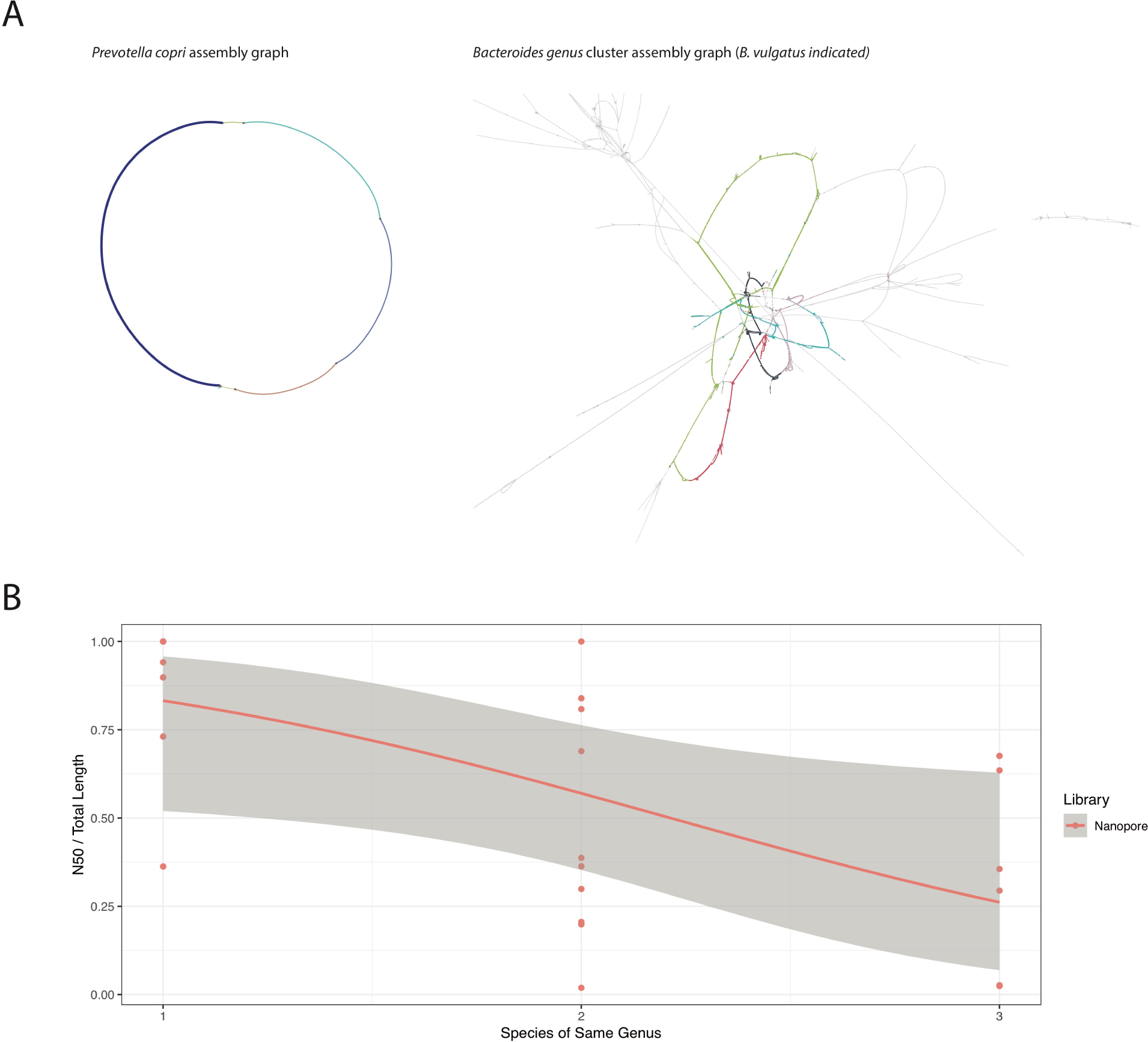

Supplement: Limitations of long read assembly. — a) Assembly graphs demonstrating uniquely assemblable sequences present in long read data. The left sequence belongs to contigs comprising the assembled genome of Prevotella copri, which is distinct enough from other organisms in the community to prevent ambiguous paths through multiple genomes. The right sequence belongs to a complex of Bacteroides genomes within which the genome of Bacteroides vulgatus is indicated in colored strands. This complex arises from a higher number of genomically similar organisms in admixture within the community. This creates a high number of ambiguous junctions in the assembly graph where multiple unique sequences can be assembled, visible as loops in this visualization. Long reads disambiguate these junctions when they are sufficiently long and well-positioned to reveal true paths through the graph, and the odds of this occurring are increased with higher raw read N50. b) Assembly contiguity, expressed as per-bin N50 divided by total bin length, as a function of total count of bins of the same genus and sample for bins from samples P1, P2-A, P2-B, and P2-coassembled that had >300x coverage, >1 Mbp total length, and ≤ 3 other bins from same genus (n = 24). As genome contiguity approaches completion, the value N50 divided by total length approaches one. With more bins from the same genus within a given community, the observed bin assembly contiguity is reduced. This is attributable to the increased likelihood of highly similar sequences occurring in multiple genomes. Line indicates fitted generalized linear model and shading indicates 95% confidence interval. [file 41587_2020_422_Fig17_ESM.jpg]
