# Supplementary material for: Complete, closed bacterial genomes from microbiomes using nanopore sequencing
Source: Nat Biotechnol. 2020 Feb 10;38(6):701–7. doi: 10.1038/s41587-020-0422-6 (PMC7283042; doi:10.1038/s41587-020-0422-6)
Supplement: Supplementary Information — Supplementary Figs. 1–14 and Notes 1 and 2. [file 41587_2020_422_MOESM1_ESM.pdf]

nature biotechnology

SUPPLEMENTARY INFORMATION
https://doi.org/10.1038/s41587-020-0422-6

In the format provided by the authors and unedited.

Complete, closed bacterial genomes from microbiomes using nanopore sequencing

Eli L. Moss[1,3], Dylan G. Maghini[1,3] and Ami S. Bhatt[1,2]✉

[1]Department of Genetics, Stanford University, Stanford, CA, USA. [2]Department of Medicine (Hematology, Blood and Marrow Transplantation), Stanford University, Stanford, CA, USA. [3]These authors contributed equally: Eli L. Moss, Dylan G. Maghini. ✉e-mail: asbhatt@stanford.edu

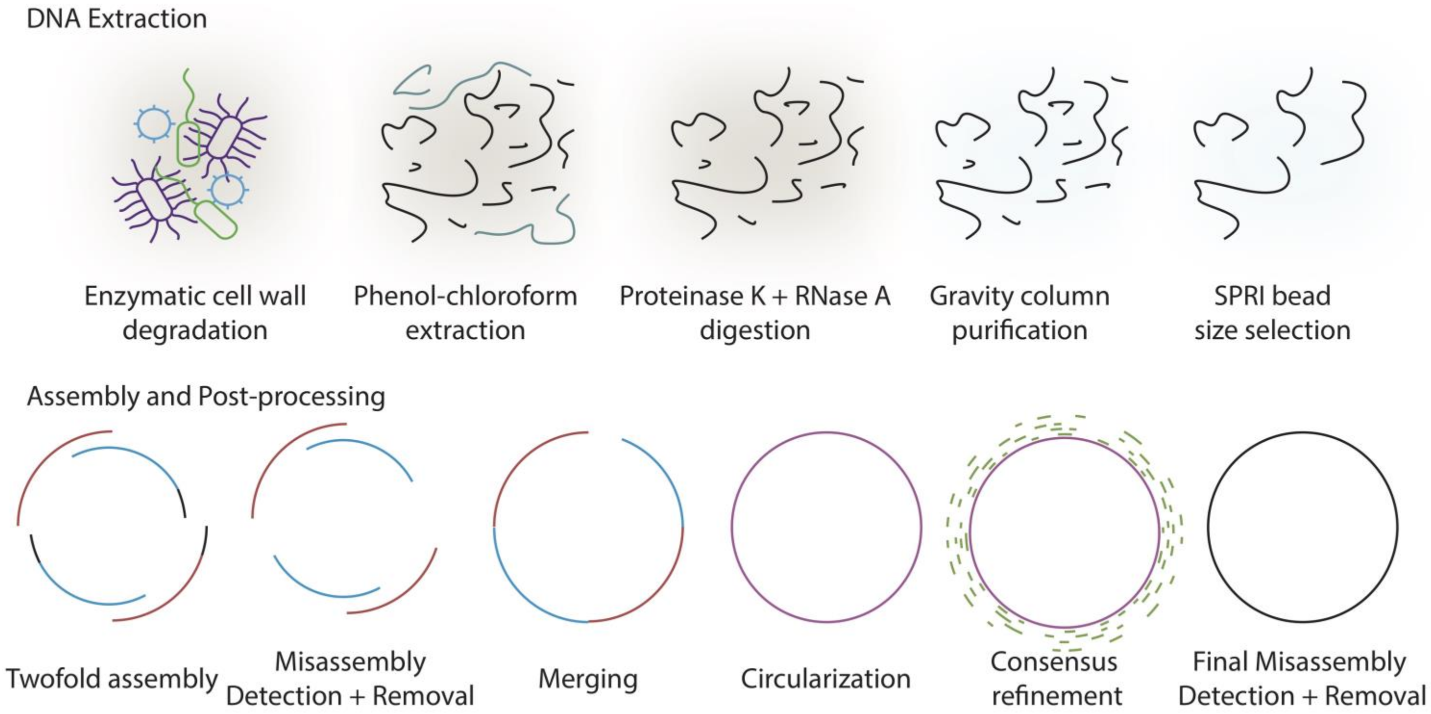

Supplementary Figure 1

Overview of the molecular and informatic workflow steps.

Extraction consists of enzymatic degradation of bacterial cell walls followed by an initial DNA extraction in phenol-chloroform. This is followed by a proteinase K and RNase A digestion at high temperature and purification with a gravity column. Finally, small fragments are removed by modified SPRI bead size selection. After sequencing and basecalling, read sequences are assembled twice with varying genomeSize parameter values. These two assemblies are screened for sites not spanned by multiple long reads indicating misassembly, merged, and then circular sequences are identified and trimmed. The consensus sequence is refined by either short-read or long-read polishing, and final assemblies are screened once more for any misassembled sites not spanned by long reads.

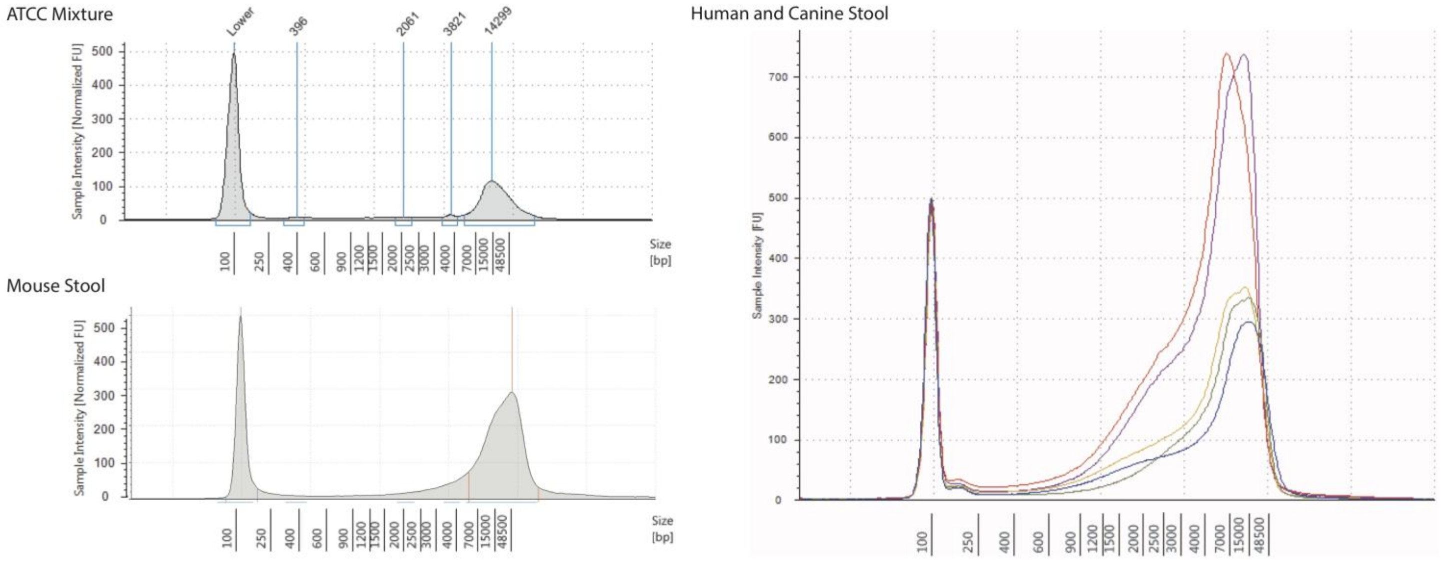

Supplementary Figure 2

TapeStation traces of a variety of stool samples.

Left: TapeStation traces of high molecular weight DNA extracted from ATCC MSA-2006 defined bacterial mixture and mouse stool. The curve demonstrates a high quantity (as measured by fluorescence units on the y-axis) in the >4000 bp regime for the extracted DNA. The peak at 100 bp represents the molecular weight marker standard. Right: TapeStation trace of high molecular weight DNA extracted from canine (blue), human stool sample not included in this study (green), healthy human P1 stool sample (red), healthy human sample P2-B stool sample (light brown), and healthy human P2-A stool sample (purple). The peak at 100 bp represents the molecular weight marker standard. Extractions were performed once per sample.

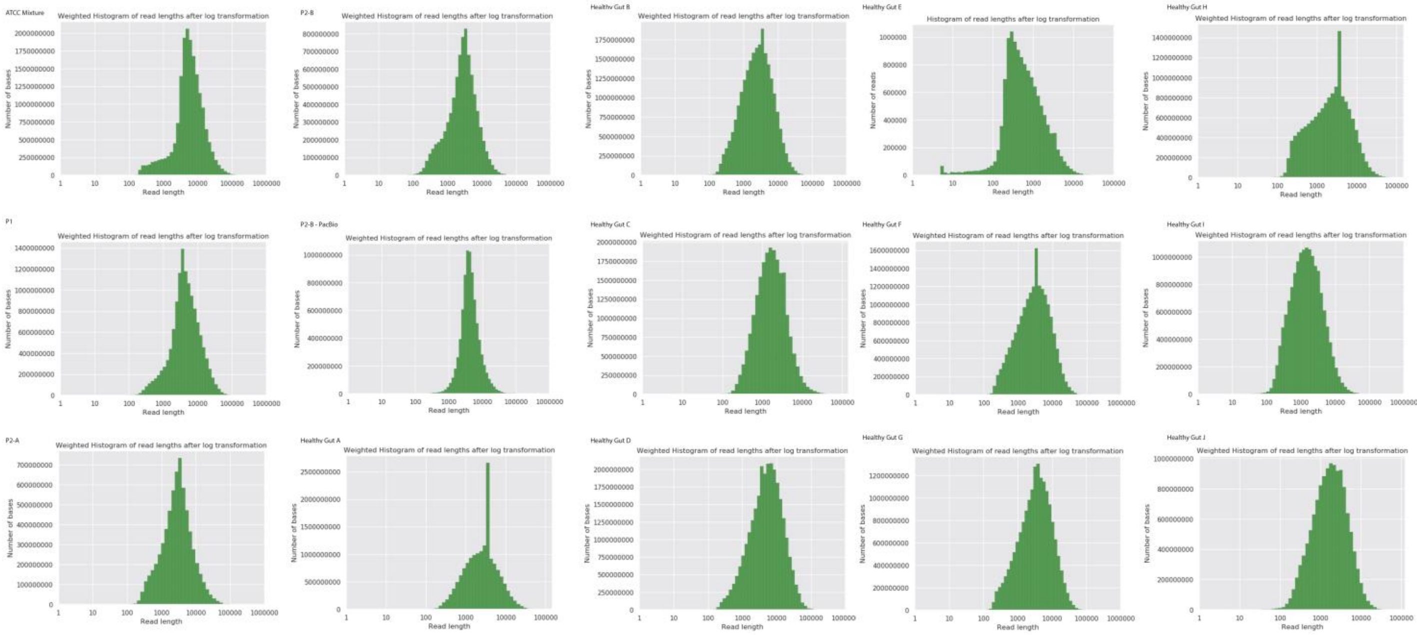

Supplementary Figure 3

Read length distributions versus total bases for all samples.

Histograms of total bases versus read length for the 13 stool samples, sequenced with the current approach, the PacBio library, and the ATCC bacterial mixture. Read lengths vary between <1 kbp to >100 kbp, with N50 values between 5 kbp and 10 kbp.

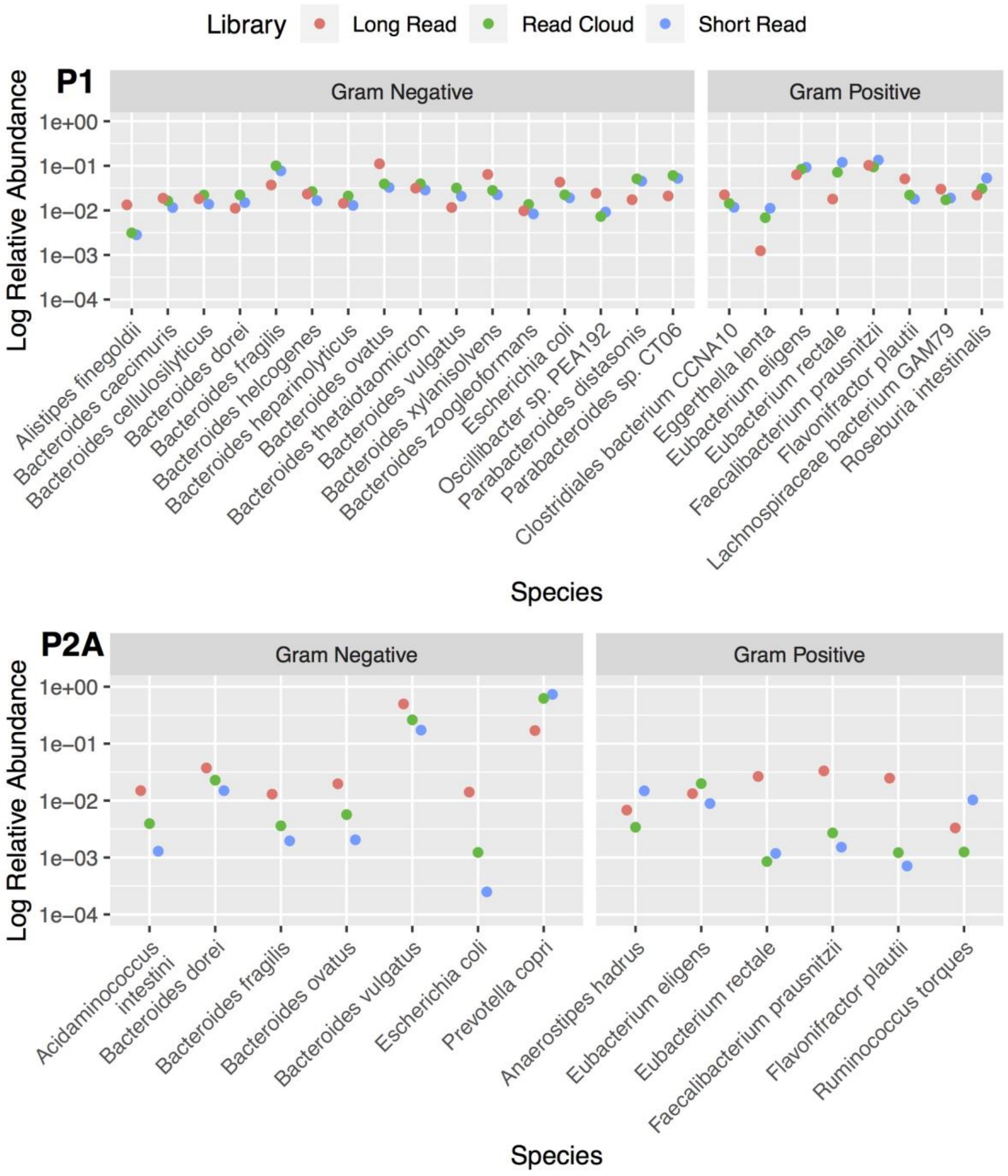

Supplementary Figure 4

Relative abundance of organisms across approaches.

Relative abundance of organisms in samples P1 and P2-A across long read, read cloud and short read libraries, stratified by Gram stain characteristics. A chief concern of bacterial lysis methods is systematic taxonomic bias, particularly with regard to cell wall structure. Although precise rank order abundances are not identical between long and short read based approaches, deviations do not assort with broad taxonomic differences in cell wall structure in the two stool samples.

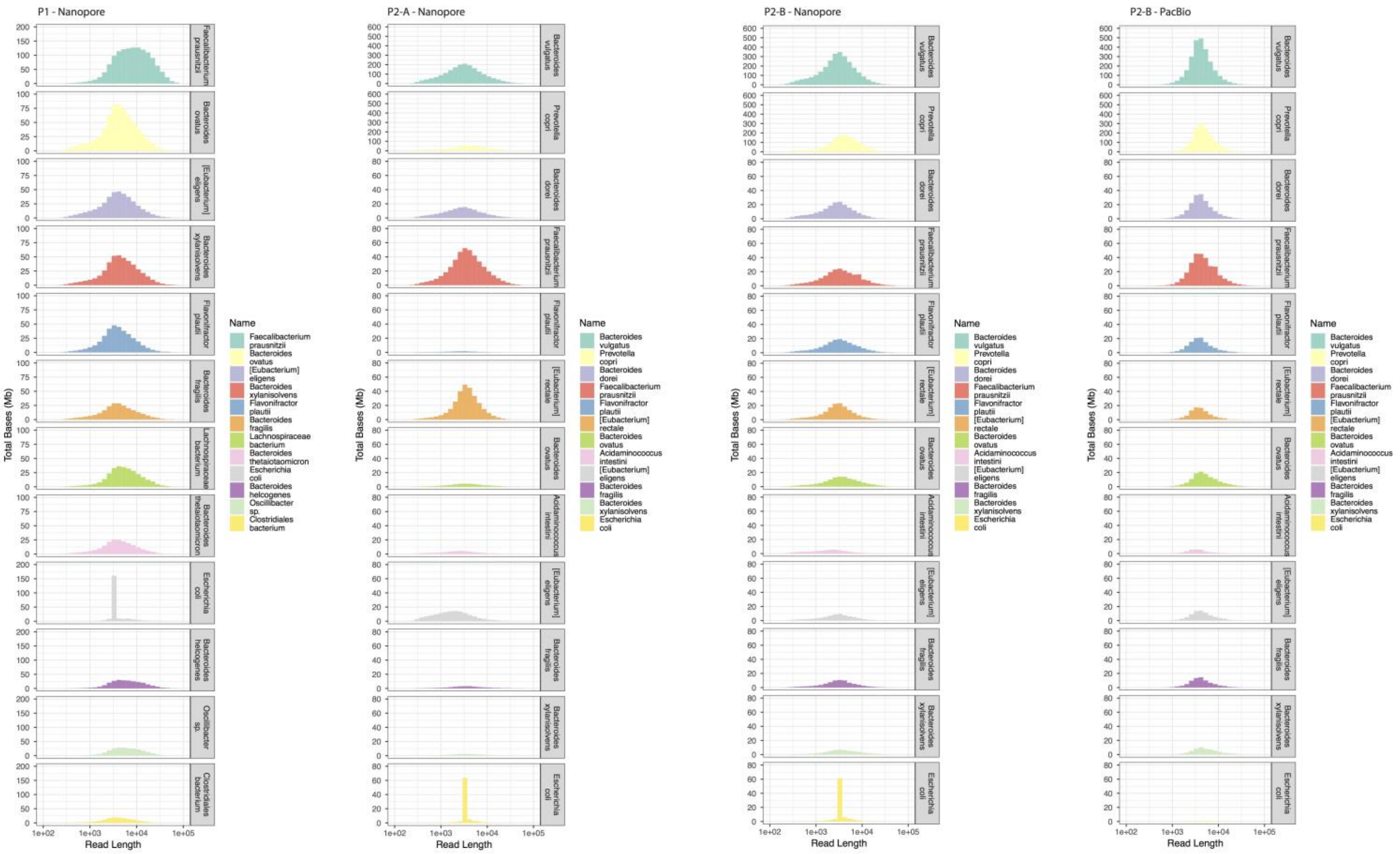

Supplementary Figure 5

Read length distributions per organism in long read sequencing from human stool samples.

Although small variations between organisms are visible, overall read length distributions are visibly more consistent in stool DNA extractions than the defined bacterial mixture. *E. coli* demonstrates a visible peak in read length distribution corresponding to reads originating from conserved sequences most likely misattributed to these organisms (see text).

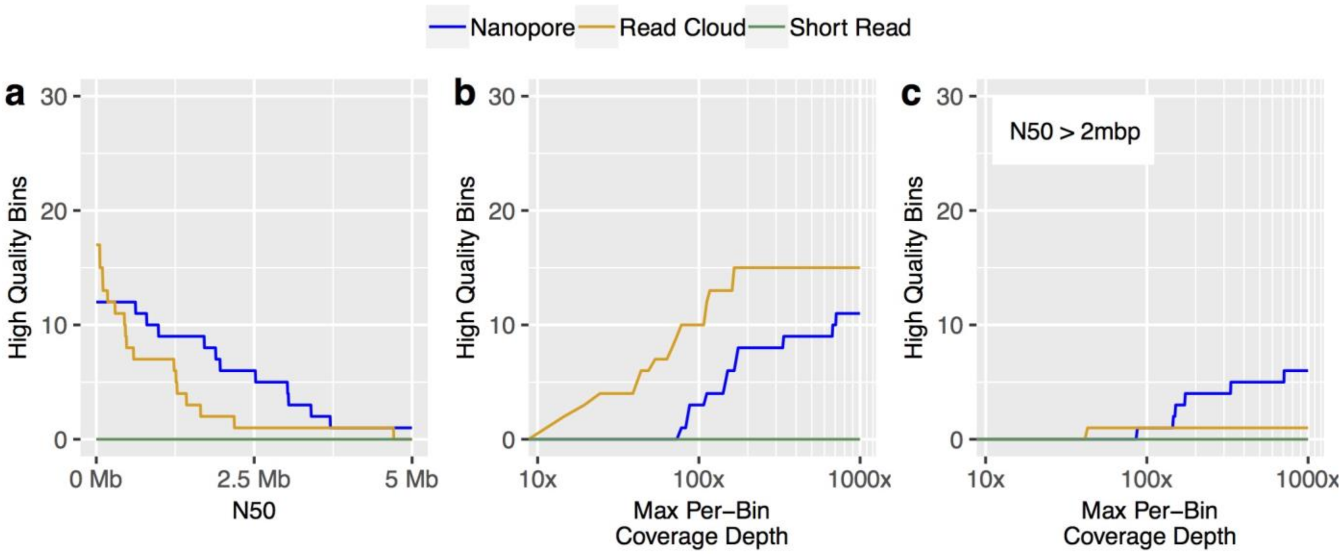

Supplementary Figure 6

Bin counts for nanopore, read cloud and short read approaches.

(a) High quality genome bins with a minimum N50. (b) High quality genome bins below a given depth of read coverage. (c) High quality genome bins with an N50 exceeding 2 Mbp below a given read coverage depth.

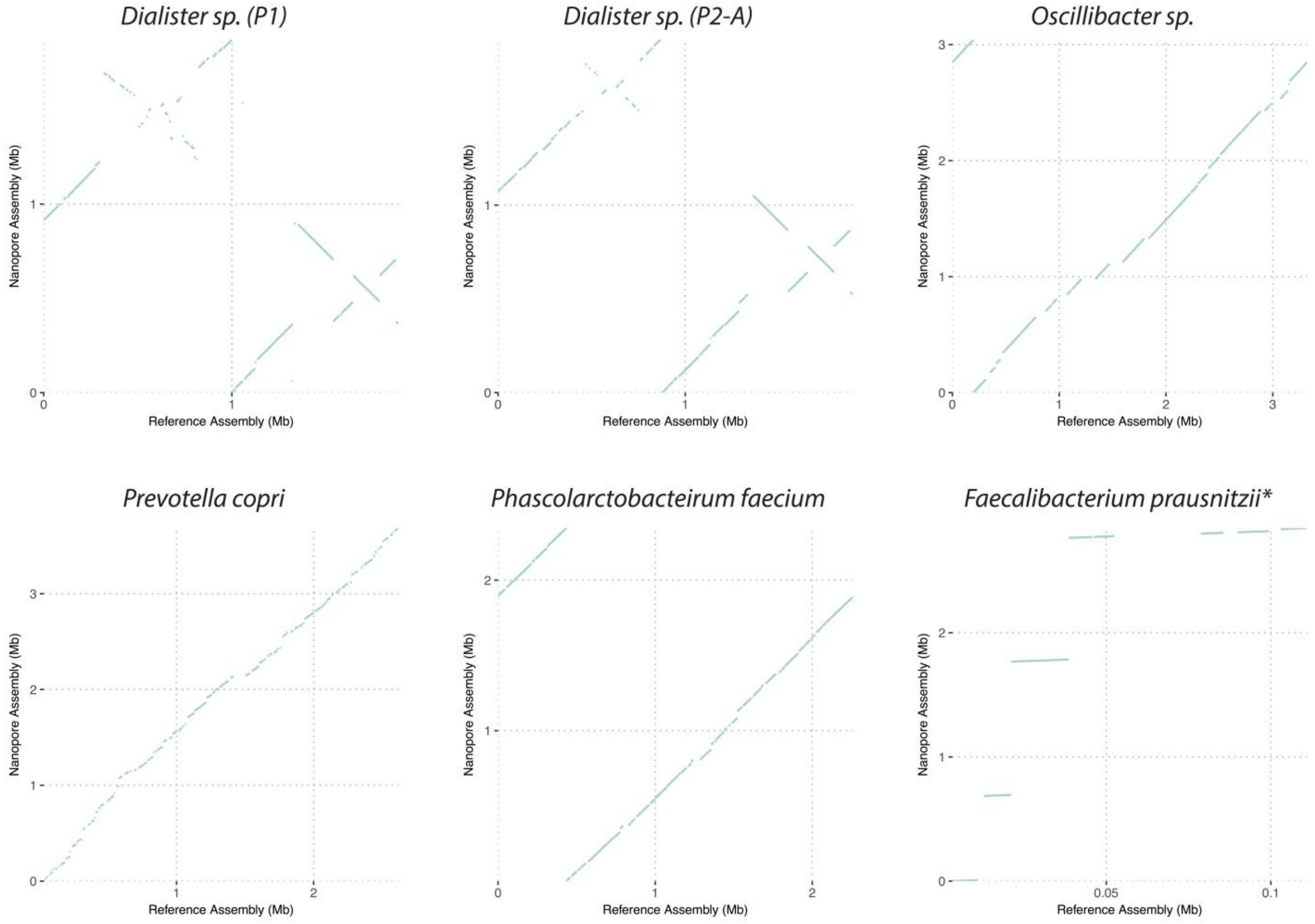

Supplementary Figure 7

Reference alignment dotplots for closed genomes obtained by nanopore long read sequencing and assembly.

Although assemblies share broad structural similarity to available references, there are cases where observed organisms are significantly structurally diverged (e.g. *Dialister*) and in one case bears minimal similarity to the closest available reference (*Faecalibacterium*; note shorter *x*-axis). Asterisks denotes genome later annotated as putative *Cibiobacter*.

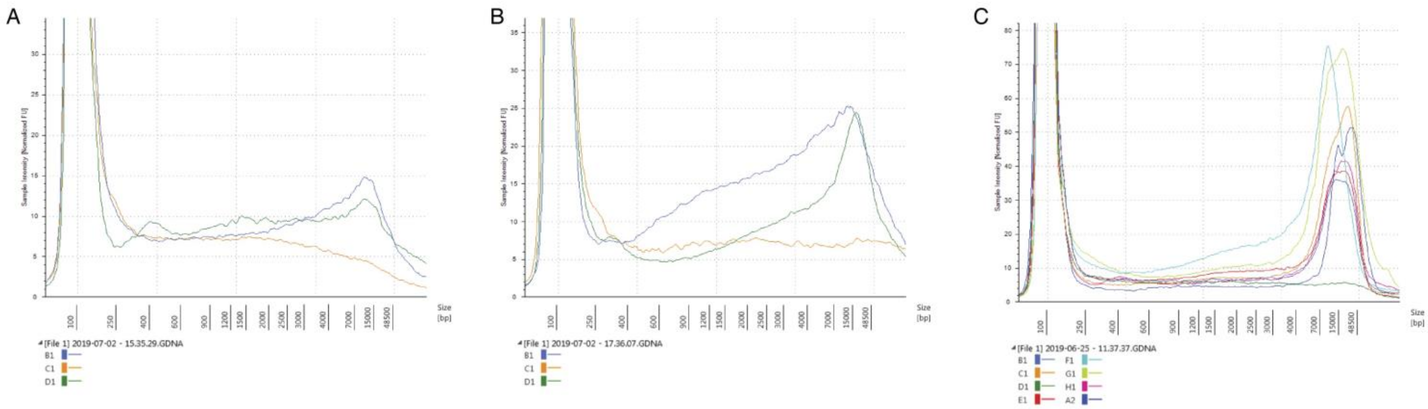

Supplementary Figure 8

TapeStation quantification of DNA fragments obtained from healthy adult samples.

(a) TapeStation quantification of DNA extracted from healthy adult stool samples A (green), C (blue), and E (yellow), prior to size selection. (b) TapeStation quantification of samples from panel A, after size selection with SPRI beads. All but one sample (A, green) yielded very short fragments and insufficient DNA after size selection. (c) TapeStation quantification of DNA extracted from eight healthy adult stool samples (A, B, C, E, F, G, H, J) after extraction with the present approach shows a significant enrichment for DNA fragments above 10 kb and minimal shorter fragments. Extractions were performed once per sample.

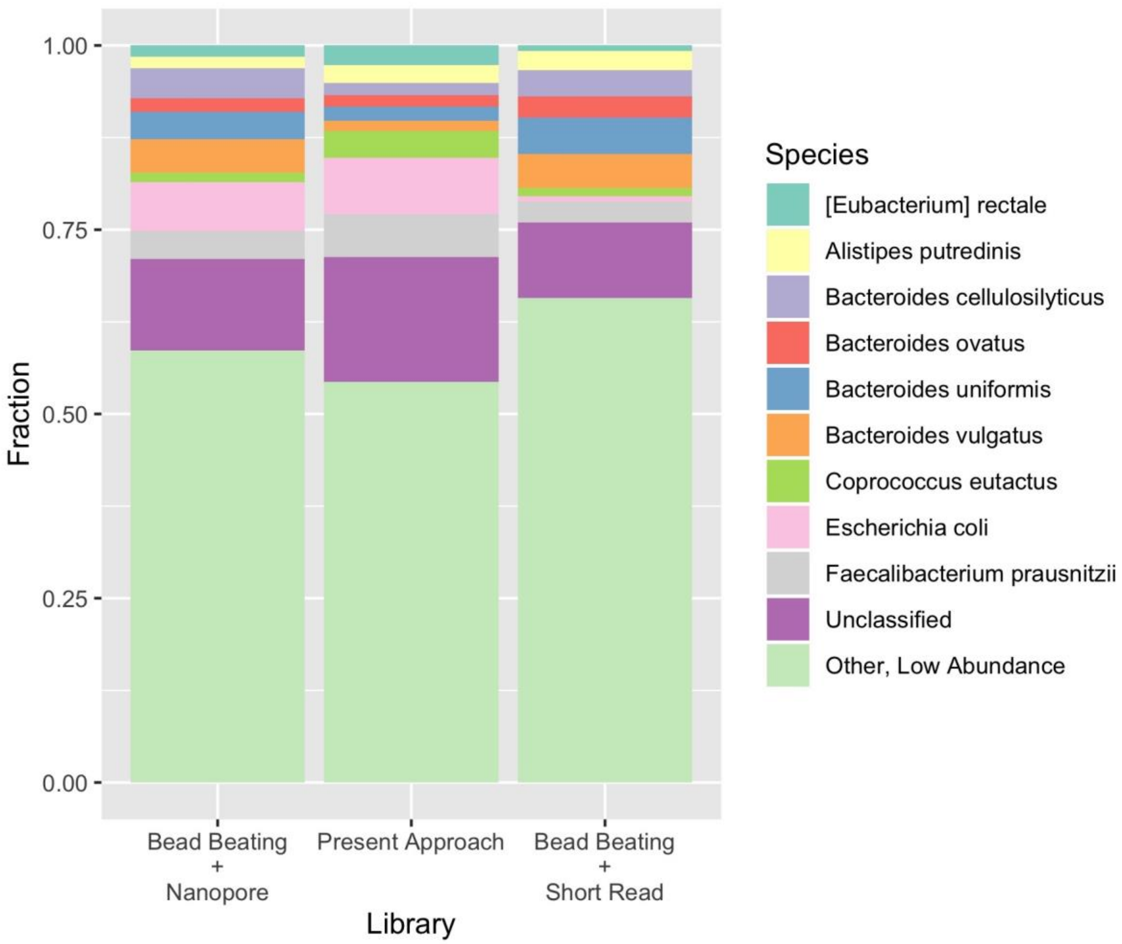

Supplementary Figure 9

Taxonomic composition across extraction and sequencing methods.

Taxonomic composition of healthy adult stool sample A, which was subjected to bead beating followed by nanopore sequencing vs. short read sequencing; and which was also subjected to the high molecular weight extraction and nanopore sequencing. Only one of the ten additional healthy adult stool samples that were bead beaten yielded sufficient quantities of SPRI size-selected DNA for subsequent nanopore sequencing. All other samples yielded short fragments by TapeStation quantification (Supplementary Figure 8). The ten most abundant taxa are depicted in this figure for clarity of representation.

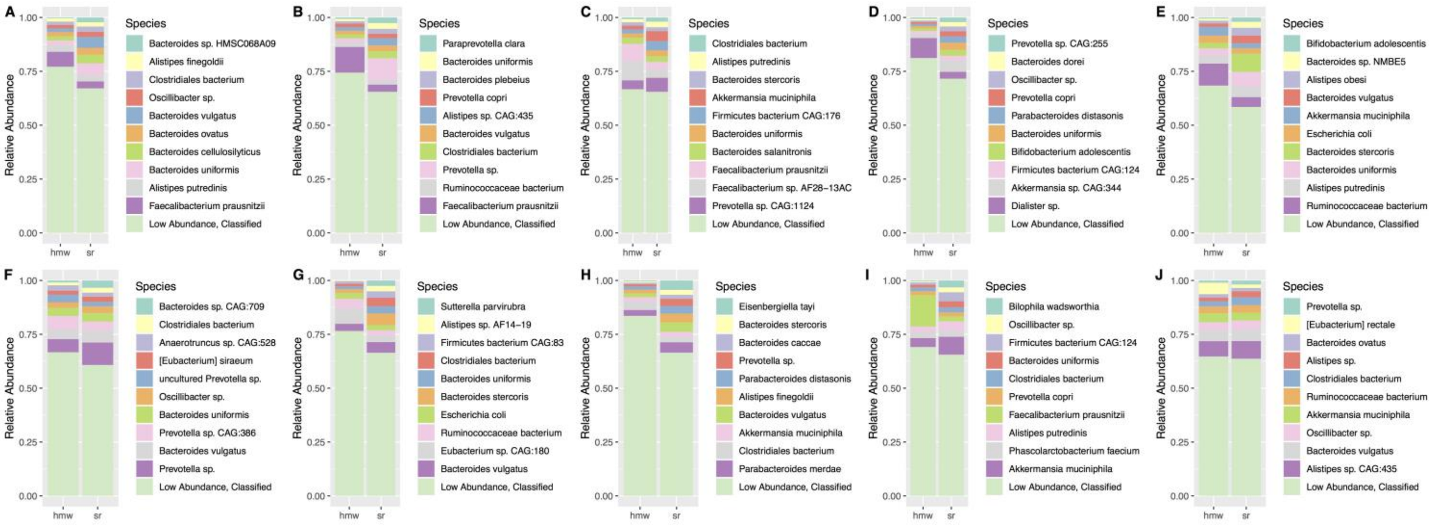

Supplementary Figure 10

Sequence-derived taxonomic composition of additional healthy adult cohort samples.

Ten additional healthy adult stool samples were subjected to both the present approach (hmw) and a conventional approach (sr) consisting of bead-beating lysis in conjunction with short read sequencing. The eleven most abundant species in short read sequencing data are shown in both libraries for visual clarity. The organisms most highly represented in the conventional approach are recovered by the present approach in all cases.

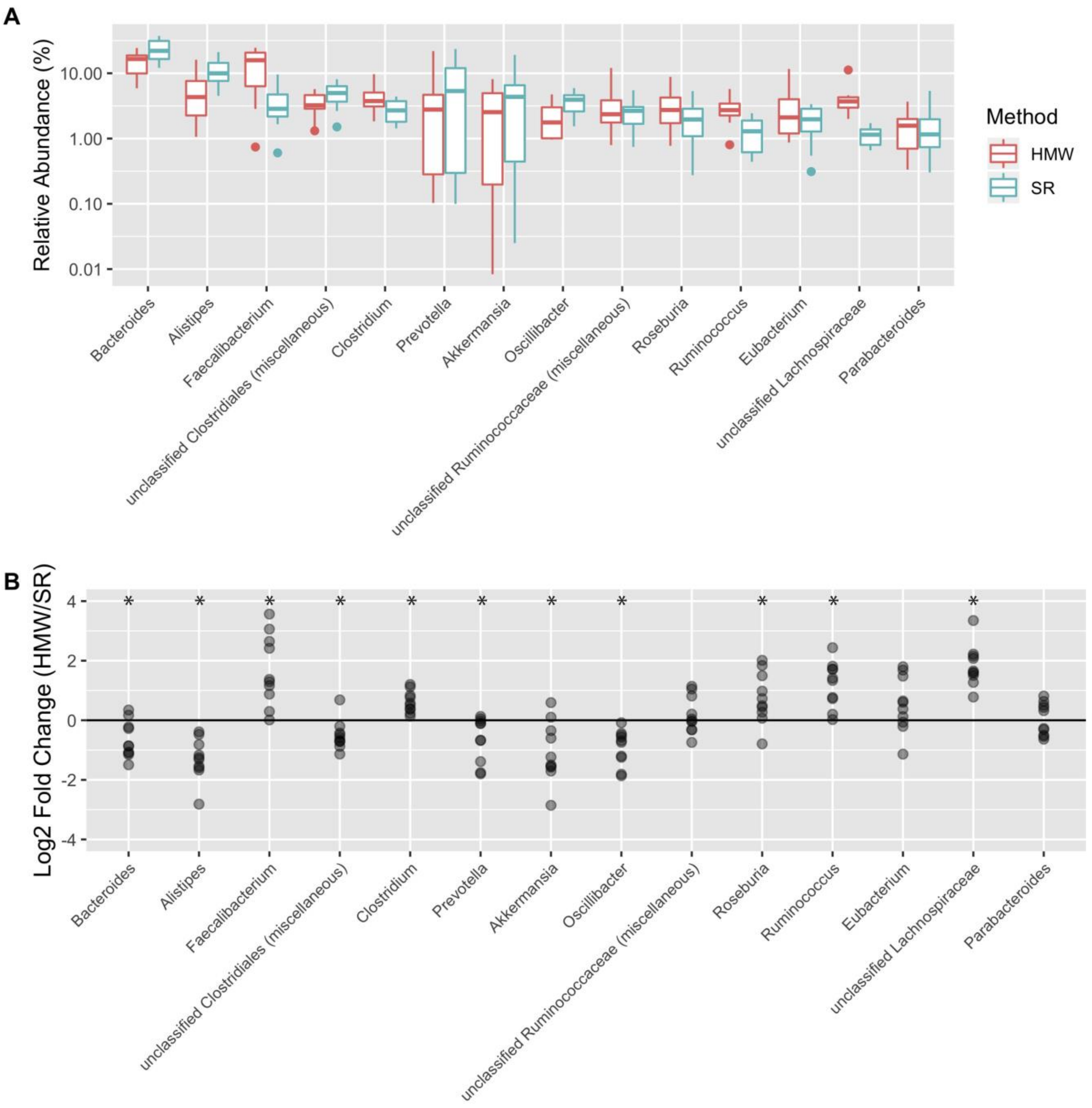

Supplementary Figure 11

Genus-level comparison of bacterial relative abundances across extraction and sequencing approaches.

A) Comparison of relative abundances of most abundant genera in healthy human stool samples (n=10) processed with bead beating and short read sequencing (SR) or the present approach and nanopore sequencing (HMW). Only genera with median relative abundances of >1% are shown for visual clarity. Boxes represent quartiles and median values, whiskers represent maximum and minimum values or quartiles ± 1.5 times the interquartile range, and points represent outliers. B) Comparison of log2 fold change of genera between samples extracted with HMW and SR approaches (n=10) demonstrates that bias for certain genera is consistent across samples. A single asterisk indicates a p-value < 0.05, two-sided Wilcoxon signed-rank test.

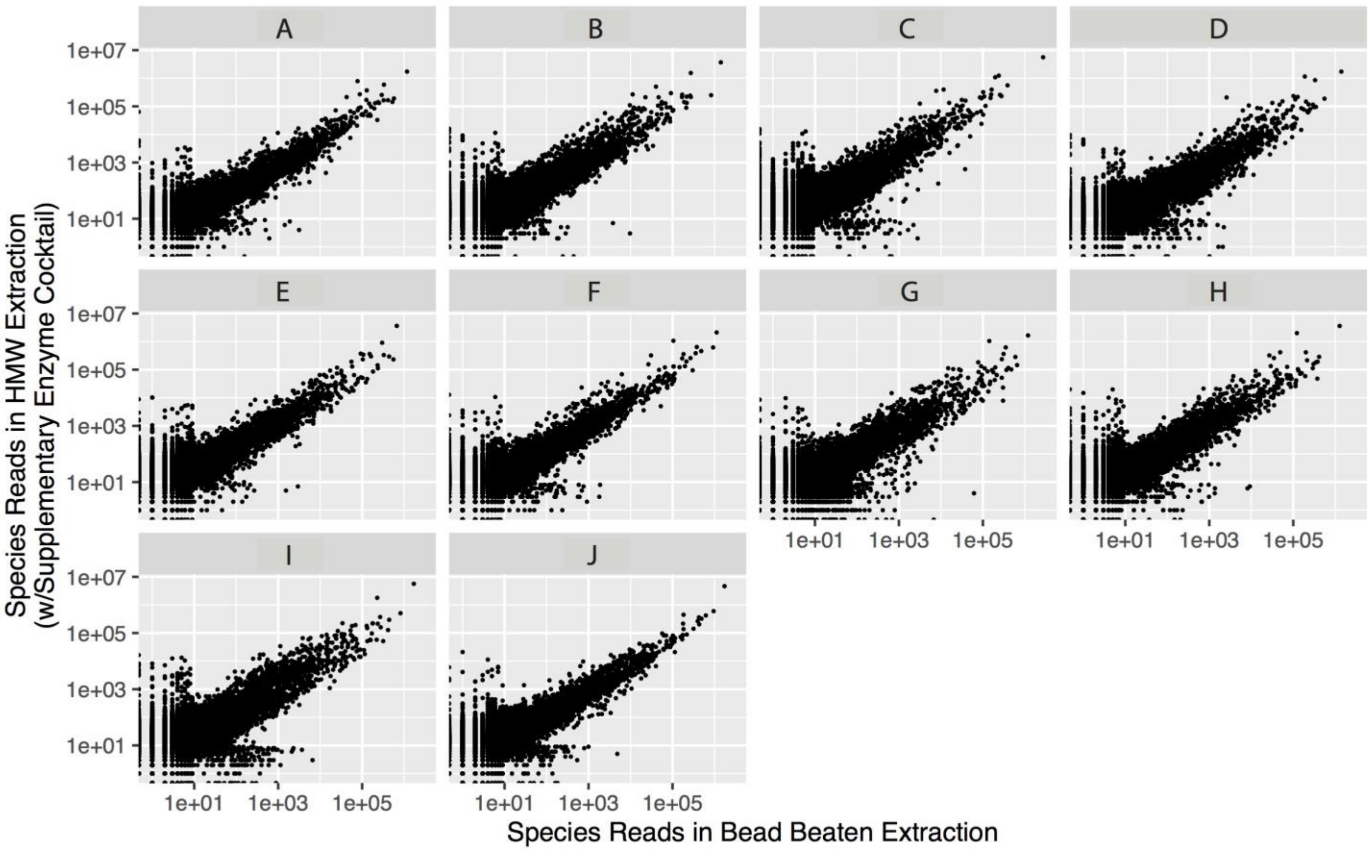

Supplementary Figure 12

Comparison of species-level read counts across extraction and sequencing approaches.

Read counts of species detected by the gold standard approach of bead beating and short read sequencing (x-axis) versus read counts of species detected by the present approach incorporating supplemental lytic enzymes (see methods) (y-axis). On the log-transformed read counts, the two approaches show a Pearson correlation of 0.79 across samples (n=10). In addition, of the 18,462 total cases in which a given species was more than tenfold enriched in relative abundance in either approach over the other, we found that our approach yielded the higher relative abundance in 95% of cases, suggesting the potential for richer taxonomic sensitivity by our method.

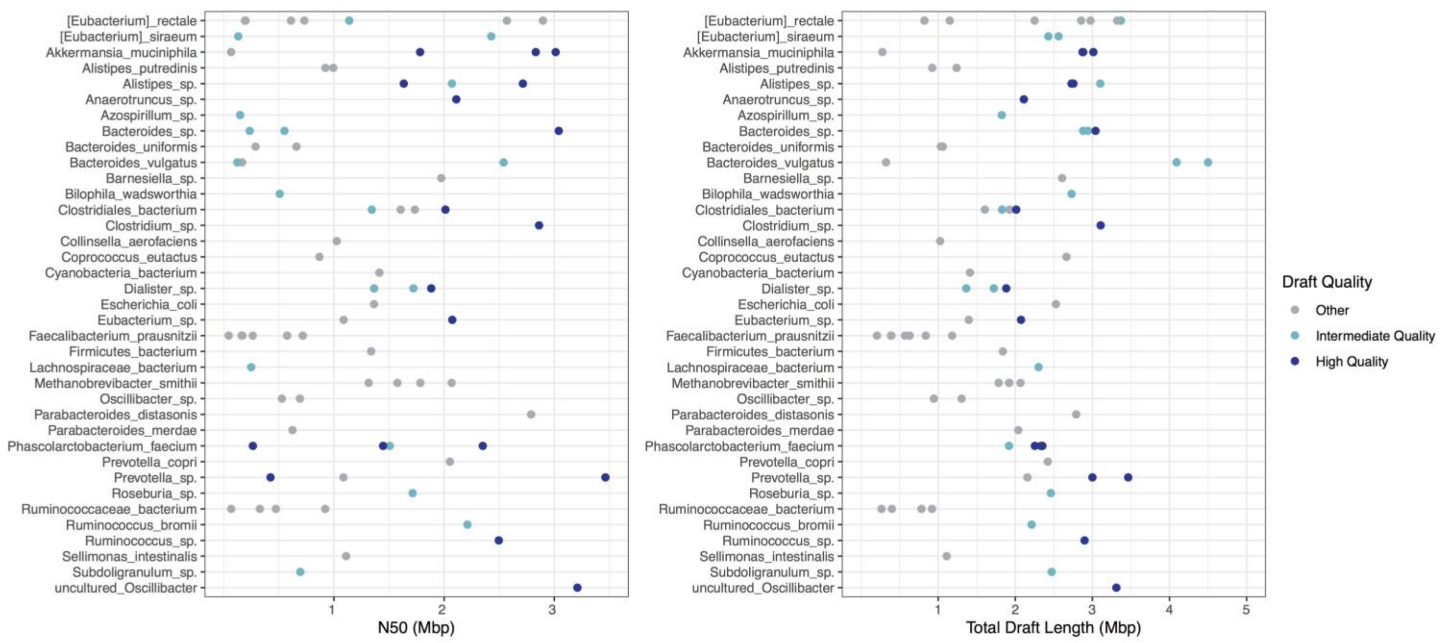

Supplementary Figure 13

Contiguity, size and quality of species genome draft sequences obtained by the present approach from ten healthy adult stool samples.

The present approach remains capable of yielding high quality (>90% completeness, <5% contamination, at least 1 each of 5S, 16S and 23S rRNA, at least 18 tRNA loci), contiguous drafts when applied to additional complex samples. Drafts are shown for all organisms with at least 2% relative abundance, at least intermediate quality (high quality with minimum completeness reduced to 75%), or an N50 of at least 1 Mbp. For each species genome draft on the y-axis, the draft N50 (left) or the total draft length (right) is shown on the x-axis. If more than one draft genome per organism was generated from the same sample, only the draft with the highest N50 is shown for clarity.

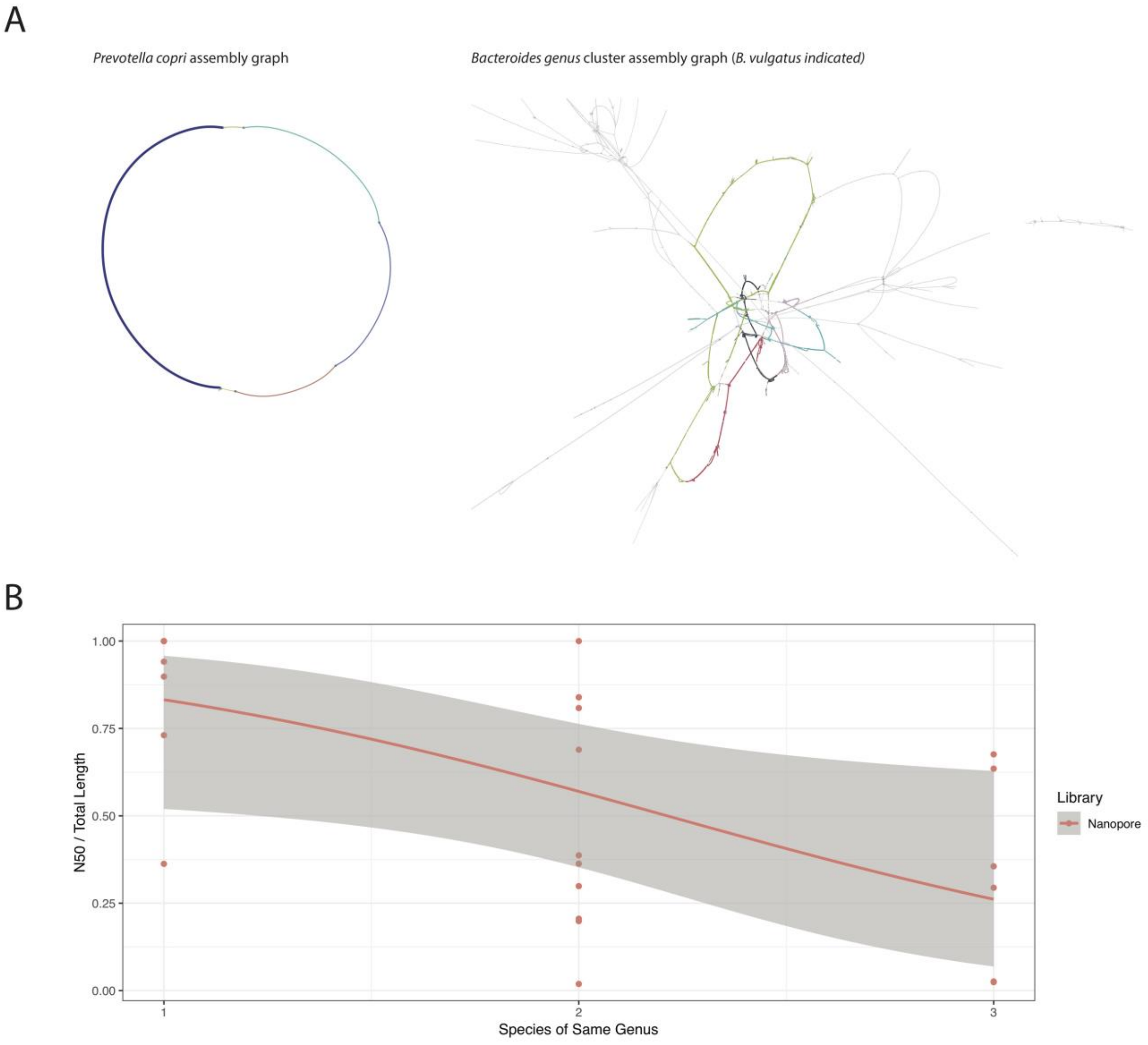

Supplementary Figure 14

Limitations of long read assembly.

A) Assembly graphs demonstrating uniquely assemblable sequences present in long read data. The left sequence belongs to contigs comprising the assembled genome of *Prevotella copri*, which is distinct enough from other organisms in the community to prevent ambiguous paths through multiple genomes. The right sequence belongs to a complex of *Bacteroides* genomes within which the genome of *Bacteroides vulgatus* is indicated in colored strands. This complex arises from a higher number of genomically similar organisms in admixture within the community. This creates a high number of ambiguous junctions in the assembly graph where multiple unique sequences can be assembled, visible as loops in this visualization. Long reads disambiguate these junctions when they are sufficiently long and well-positioned to reveal true paths through the graph, and the odds of this occurring are increased with higher raw read N50. B) Assembly contiguity, expressed as per-bin N50 divided by total bin length, as a function of total count of bins of the same genus and sample for bins from samples P1, P2-A, P2-B, and P2-coassembled that had >300x coverage, >1 Mbp total length, and ≤ 3 other bins from same genus (n = 24). As genome contiguity approaches completion, the value N50 divided by total length approaches one. With more bins from the same genus within a given community, the observed bin assembly contiguity is reduced. This is attributable to the increased likelihood of highly similar sequences occurring in multiple genomes. Line indicates fitted generalized linear model and shading indicates 95% confidence interval.

Supplementary Notes

Generating closed bacterial genomes from nanopore sequencing of complex metagenomes

Eli L. Moss, Dylan G. Maghini, Ami S. Bhatt

Table of Contents

1. Consensus Refinement and Homopolymer Error
2. PacBio and Nanopore Comparison

Consensus Refinement and Homopolymer Error

After assembly, Lathe performs consensus refinement, which aims to correct homopolymer and mismatch errors in nanopore sequencing, artifacts which can affect gene prediction if left uncorrected[1]. To evaluate the nucleotide accuracy of our approach, we used MetaQuast[2] to compare refined assemblies to the available closed reference genome sequences. Prior to consensus refinement, the assembly contained 494 indels and 88 mismatches per 100 kbp. Consensus refinement with short reads removed 93% of indels and 82% of mismatches, leaving 35 and 16 per 100 kbp, respectively. Although short read polishing is effective in removing errors from the raw assembly, this method of error correction is dependent on uniform coverage of the long read assembly with short reads; 1% of the nanopore assembly did not receive sufficient short read coverage for consensus refinement, leaving on average 5 indels per 100kbp uncorrected. Consensus refinement with long reads removed 83% of indels and 71% of mismatches across the whole assembly. Combining long and short read consensus refinement removes an additional 3% of indels over short read refinement alone, but incurs considerably higher computational cost and does not improve mismatch correction when short read coverage depth is uniform. Thus short read consensus refinement alone was selected for the default Lathe workflow. However, we have included the option of combining both forms of correction for cases of sparse short read coverage where some areas would otherwise be left entirely uncorrected. By contrast, direct assembly of short reads produced an assembly containing on average 2 indels and 19 mismatches per 100 kbp, although at much-reduced contiguity (Supplementary Table 3). Although long read-based consensus refinement is highly effective, we find that it cannot yet fully replace short read correction. Both methods are incorporated into Lathe, with additional parallelization and aggregation steps needed to allow application of the current gold-standard short read polishing tool to metagenomic-scale datasets.

A major contributor to the relatively high indel error rate of long read assembly is homopolymer error, the tendency of the nanopore sequencing approach to incorrectly call the length of homopolymeric repeat sequences. In the ATCC mixture, we found that uncorrected long read assembly demonstrated a 0.7% error rate in 3-mer homopolymers, assembled too short by an average of 0.3 nucleotides. This worsens to a 41% error rate on 7-mer homopolymers, which were assembled too short by an average of 1.1 nucleotides. On average, 88 homopolymers of length 3 or greater were found per kilobase in the uncorrected assembly, of which on average 2.6 (3%) were found to require correction with short reads. When short

reads are unavailable, Lathe reverts to long read consensus refinement and yields structurally correct and complete genomes, although with reduced nucleotide accuracy. Recent advances in nanopore sequencing technology have decreased homopolymer error and will likely continue to do so, lessening or removing the need for supplemental short read sequencing to achieve genomes with high nucleotide fidelity.

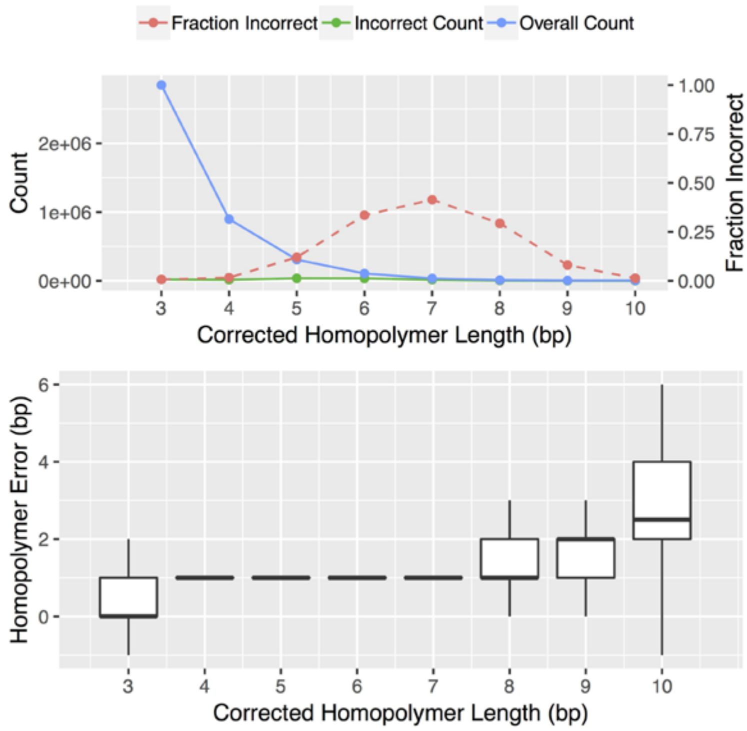

Homopolymer count as a function of length, and homopolymer error in assembled sequence as a function of length in ATCC assembly (n = 4,213,331 total homopolymers). We found that uncorrected long read assembly demonstrated a 0.7% error rate with 3-mer homopolymers, assembled too short by an average of 0.3 nucleotides. This worsens to a 41% error rate on 7-mer homopolymers, which were assembled too short by an average of 1.1 nucleotides. On average, 88 homopolymers of length 3 or greater were found per kilobase of assembled sequence, of which 2.6 (3%) were found to require correction with short reads. Boxes represent quartiles and median values, whiskers represent maximum and minimum values or quartiles ± 1.5 times the interquartile range.

PacBio and Nanopore Comparison

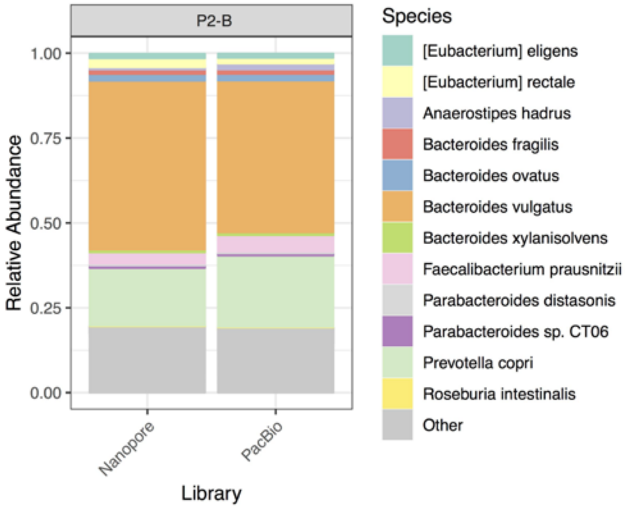

Taxonomic read composition for nanopore and PacBio sequencing from sample P2-B. Relative abundances of organisms classified in long reads from nanopore and PacBio sequencing are shown on the y-axis.

To compare our nanopore-based long read assembly approach to data generated by PacBio long read sequencing, we subjected HMW DNA extracted from sample P2-B to PacBio library preparation and sequencing with a single SMRT cell on a Sequel sequencing instrument, followed by processing with Lathe. The PacBio sequencing run produced 2- to 4-fold more raw read data than the tested nanopore flowcells, so the PacBio data were randomly downsampled to the same total dataset size to maintain comparability. Although overall data yield was fourfold higher from PacBio, the higher cost of sequencing resulted in a 1.5-fold higher cost per base compared to nanopore (Supplementary Table 6). The taxonomic composition of PacBio long reads was highly similar to those obtained from nanopore sequencing. The read lengths obtained by PacBio Sequel sequencing were higher than those obtained by nanopore sequencing (4.1 kbp vs 2.9 kbp raw read N50), yet the assembly produced was far more fragmentary than that produced by Lathe-processed nanopore data (N50 25 kbp vs. 198 kbp), and no larger in total size (84 mbp vs. 85 mbp) (Supplementary Table 5). This is not attributable to average read length (Supplementary Figure 3), species-specific read length (Supplementary Figure 5), or data volume (Supplementary Table 2), which are all slightly higher in the PacBio dataset. Instead, as expected we found coverage from PacBio sequencing to occur in a distinctly stepwise fashion due to circular consensus sequencing. This results in more widely varied coverage than nanopore data, leaving gaps of zero coverage even in heavily covered regions. In all, we found 7,630 zero-coverage gaps of 2 base pairs or more in PacBio data aligned to the nanopore-assembled draft sequences. Regions receiving uninterrupted read coverage from PacBio sequencing assembled with equal effectiveness to nanopore sequencing,

demonstrated by a full-length draft of *Prevotella copri* and a high quality but incompletely assembled draft of *Phascolarctobacterium faecium* obtained from PacBio sequencing. As noted previously, this is likely an effect of the mechanism of PacBio sequencing, which repeatedly sequences template molecules, thus increasing consensus read accuracy but decreasing evenness of coverage.

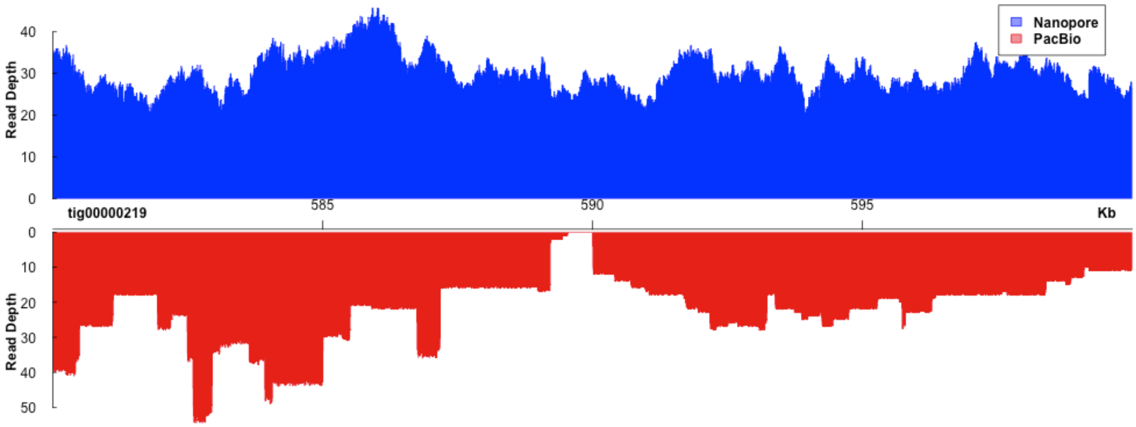

Coverage depth histograms across 20 kb of a nanopore-assembled bacterial genome from nanopore and PacBio long reads. The nanopore data distribution is characterized by small, gradual variations in local read depth coverage of approximately ± 10X in magnitude. PacBio data have a distinctly stepwise depth distribution, attributable to circular consensus sequencing, which generates a larger range of coverage, dropping to zero in the center of the plot. This inconsistency leaves a large quantity of coverage gaps, leading to a fragmentary assembly.

References

1. Watson, M. & Warr, A. Errors in long-read assemblies can critically affect protein prediction. *Nat. Biotechnol.* **37**, 124–126 (2019).
2. Mikheenko, A., Saveliev, V. & Gurevich, A. MetaQUAST: evaluation of metagenome assemblies. *Bioinformatics* btv697 (2015).
