# Supplementary material for: Complete, closed bacterial genomes from microbiomes using nanopore sequencing
Source: Nat Biotechnol. 2020 Feb 10;38(6):701–7. doi: 10.1038/s41587-020-0422-6 (PMC7283042; doi:10.1038/s41587-020-0422-6)
Supplement: Supplementary file 16 — Reporting Summary [file 41587_2020_422_MOESM2_ESM.pdf]

nature research

Corresponding author(s): Ami S. Bhatt

Last updated by author(s): Dec 10, 2019

Reporting Summary

Nature Research wishes to improve the reproducibility of the work that we publish. This form provides structure for consistency and transparency in reporting. For further information on Nature Research policies, see Authors & Referees and the Editorial Policy Checklist.

Statistics

For all statistical analyses, confirm that the following items are present in the figure legend, table legend, main text, or Methods section.

n/a	Confirmed	
☐	☒	The exact sample size (*n*) for each experimental group/condition, given as a discrete number and unit of measurement
☐	☒	A statement on whether measurements were taken from distinct samples or whether the same sample was measured repeatedly
☐	☒	The statistical test(s) used AND whether they are one- or two-sided<br>*Only common tests should be described solely by name; describe more complex techniques in the Methods section.*
☒	☐	A description of all covariates tested
☒	☐	A description of any assumptions or corrections, such as tests of normality and adjustment for multiple comparisons
☒	☐	A full description of the statistical parameters including central tendency (e.g. means) or other basic estimates (e.g. regression coefficient) AND variation (e.g. standard deviation) or associated estimates of uncertainty (e.g. confidence intervals)
☐	☒	For null hypothesis testing, the test statistic (e.g. *F*, *t*, *r*) with confidence intervals, effect sizes, degrees of freedom and *P* value noted<br>*Give P values as exact values whenever suitable.*
☒	☐	For Bayesian analysis, information on the choice of priors and Markov chain Monte Carlo settings
☒	☐	For hierarchical and complex designs, identification of the appropriate level for tests and full reporting of outcomes
☐	☒	Estimates of effect sizes (e.g. Cohen's *d*, Pearson's *r*), indicating how they were calculated

Our web collection on statistics for biologists contains articles on many of the points above.

Software and code

Policy information about availability of computer code

Data collection	MinKNOW v2.2.12, Guppy v2.3.5 were used to collect and base-call collected DNA sequencing data from the ONT MinION platform.
Data analysis	BLAST v2.9.0, Canu v1.8, Pilon v1.22, CheckM v1.0.12, Racon v1.3.2, Medaka v0.6.1, mummer v3.23, miniasm v0.2, SPAdes v3.13.0, hybridSPAdes v3.13.0, wtdbg2 v2.2, Ra v0.2.1, Flye v2.4.2, quickmerge v0.40, OPERA-MS, Quast v5.0.0, Prokka v1.13.3, Barrnap v0.9, nucmer v3.1, bcftools v1.9-107, ggplot2 v3.2.1, PHASTER, 2.0.8, and Snakemake v5.4.0 were used to analyze the data used in this study. In addition, custom workflows and scripts used in this study can be found at https://github.com/elimoss/metagenomics_workflows.

For manuscripts utilizing custom algorithms or software that are central to the research but not yet described in published literature, software must be made available to editors/reviewers. We strongly encourage code deposition in a community repository (e.g. GitHub). See the Nature Research guidelines for submitting code & software for further information.

Data

Policy information about availability of data

All manuscripts must include a data availability statement. This statement should provide the following information, where applicable:

- Accession codes, unique identifiers, or web links for publicly available datasets
- A list of figures that have associated raw data
- A description of any restrictions on data availability

All sequence data, whole metagenome assemblies and individual completed genomes can be found at NCBI BioProject under accession PRJNA508395.

Field-specific reporting

Please select the one below that is the best fit for your research. If you are not sure, read the appropriate sections before making your selection.

☒ Life sciences ☐ Behavioural & social sciences ☐ Ecological, evolutionary & environmental sciences

For a reference copy of the document with all sections, see nature.com/documents/nr-reporting-summary-flat.pdf

Life sciences study design

All studies must disclose on these points even when the disclosure is negative.

Sample size	No sample size calculation was performed, as this study examines DNA sequencing and assembly methods on previously published comparison materials.
Data exclusions	No data were excluded from analysis
Replication	The analytical approaches used in this paper are entirely deterministic, and the same assembly result can be obtained from the input data by re-running the published workflow.
Randomization	There are no experimental groups in this study, so no randomization was needed.
Blinding	There are no experimental groups in this study, so no blinding was needed.

Reporting for specific materials, systems and methods

We require information from authors about some types of materials, experimental systems and methods used in many studies. Here, indicate whether each material, system or method listed is relevant to your study. If you are not sure if a list item applies to your research, read the appropriate section before selecting a response.

Materials & experimental systems

n/a	Involved in the study	
☒	☐	Antibodies
☒	☐	Eukaryotic cell lines
☒	☐	Palaeontology
☐	☒	Animals and other organisms
☐	☒	Human research participants
☒	☐	Clinical data

Methods

n/a	Involved in the study	
☒	☐	ChIP-seq
☒	☐	Flow cytometry
☒	☐	MRI-based neuroimaging

Animals and other organisms

Policy information about studies involving animals; ARRIVE guidelines recommended for reporting animal research

Laboratory animals	Stool samples from a mouse were used in this study. The mouse samples were provided by Patrick Neuhoefer from the laboratory of Steven Artandi.
Wild animals	None
Field-collected samples	Discarded stool samples were collected from a domestic house-pet dog for this study.
Ethics oversight	All mouse samples were collected in accordance with an approved animal use protocol (Stanford University) overseen by the Stanford APLAC (Laboratory Animal Care) committee. The house-pet dog stool samples were discarded samples, the animal was put at minimal risk by this sample collection and thus did not require ethics approval for use.

Note that full information on the approval of the study protocol must also be provided in the manuscript.

Human research participants

Policy information about studies involving human research participants

Population characteristics	Adult participants were recruited from at Stanford University under one of two approved IRB protocols. Because this is a sequencing method development study, no covariates were used for selection.
Recruitment	Human subjects were invited to participate in one of two observational biobanking studies through informational flyers that were posted in various public places. Interested volunteers were then consented by the PI or study staff of one of the two

studies for participation in this study. The advertising flyers, protocol, and consent documents were all approved by the Stanford IRB (Study #42043; PI: Bhatt; Study #33727, PI: Henderson)

Ethics oversight	Stanford Institutional Review Board

Note that full information on the approval of the study protocol must also be provided in the manuscript.
